# Short-term cognitive fatigue effect on auditory temporal order judgments

**DOI:** 10.1007/s00221-019-05712-x

**Published:** 2020-01-03

**Authors:** Júlia Simon, Endre Takács, Gábor Orosz, Borbála Berki, István Winkler

**Affiliations:** 1 Institute of Cognitive Neuroscience and Psychology, Research Centre for Natural Sciences, Magyar Tudósok Körútja 2, Budapest, 1117 Hungary; 2 Department of Cognitive Science, Faculty of Natural Sciences, Budapest University of Technology and Economics, Budapest, Hungary; 3 Faculty of Humanities and Social Sciences, Pázmány Péter Catholic University, Budapest, Hungary; 4 Institute of Psychology, Eötvös Loránd University, Budapest, Hungary; 5 Doctoral School of Psychology, Eötvös Loránd University, Budapest, Hungary; 6 Department of Psychology, Stanford University, Stanford, CA USA; 7 Multidisciplinary Doctoral School of Engineering Sciences, Széchenyi István University, Győr, Hungary

**Keywords:** Fatigue, Performance deterioration, Temporal order judgment, Subjective fatigue, Feedback valence

## Abstract

Fatigue is a core symptom in many psychological disorders and it can strongly influence everyday productivity. As fatigue effects have been typically demonstrated after long hours of time on task, it was surprising that in a previous study, we accidentally found a decline of temporal order judgment (TOJ) performance within 5–8 min. After replicating prior relevant findings we tested whether pauses and/or feedback relating the participant’s performance to some “standard” can eliminate or reduce this short-term performance decline. We also assessed whether the performance decline is specific to the processes evoked by the TOJ task or it is a product of either general inattentiveness or the lack of willingness to thoroughly follow the task instructions. We found that both feedback and introducing pauses between successive measurements can largely reduce the performance decline, and that these two manipulations likely mobilize overlapping capacities. Performance decline was not present in a similar task when controlling for the TOJ threshold and it was not a result of uncooperative behavior. Therefore, we conclude that the TOJ threshold decline is either specific to temporal processing in general or to the TOJ task employed in the study. Overall, the results are compatible with the notion that the decline of TOJ threshold with repeated measures represents a short-term cognitive fatigue effect. This objective fatigue measure did not correlate with subjective fatigue. The latter was rather related to perceived difficulty/effort, the reduction of positive affectivity, heightened sensitivity to criticism, and the best TOJ threshold.

**Electronic supplementary material:**

The online version of this article (10.1007/s00221-019-05712-x) contains supplementary material, which is available to authorized users.

## Introduction

Fatigue is one of the most common symptoms of patients seeking professional care (Stadje et al. 2016; Menting et al. 2018) and common cause of lost working hours (Ricci et al 2007; Deligkaris et al. 2014). Yet, we understand little of its underlying mechanisms (Gergelyfi et al. 2015). Fatigue experiments usually require long hours of testing and the effect sizes are often rather small (Takács et al. 2019), which may contribute to the problem of revealing the underlying mechanisms. Here we describe a paradigm that allows relatively quick detection and examination of performance deterioration.

### Fatigue and boredom

Fatigue is still lacking a clear or standardized scientific definition (Hornsby et al. 2016; Åkerstedt et al. 2004; Chalder et al. 1993). The common ground seems to be that it is a subjectively negative, although adaptive psychophysiological sensation caused by exertion/effort (Philips 2015). Contrary to the once popular energy conservation metaphor, current lines of research assume that fatigue is not a sign of resource depletion rather than an adaptive signal that arises when there is a conflict between current and alternative goals (Hockey 2011). Fatigue can manifest in performance decline (cognitive fatigue or muscle fatigue depending on the type of the task), in subjective reports, or in both (Ackerman and Kanfer 2009). The two measures do not necessarily correlate with each other (Ackerman and Kanfer 2009; Leavitt and DeLuca 2010; Gergelyfi et al. 2015; Hornsby et al. 2016; Takács et al. 2019), but correlation between them has been found in some studies (Moore et al. 2017; Hopstaken et al. 2015b). The lack of close correlation between the two can be explained by a two-choice strategy: perception of difficulty can lead to increased effort to maintain performance or to goal aspiration reduction (Hockey 2011). Another possible source of discrepancy could be that fatigue is a “feeling” and it is by definition a result of a cognitive evaluation: The same physiological state might be interpreted differently depending on someone’s perceived control, effectivity, expectancies, or the individual’s evaluation of task difficulty or even what one considers fatigue.

Boredom can also lead to performance decline and can be defined as an “emotional cue that one needs to pursue a goal different from what one is currently pursuing” (Raffaelli et al. 2018). Note that this is the same function as was also proposed as possible source of fatigue. Boredom’s distinctive features are proposed to be unpleasantness and that it is accompanied by low arousal (Vogel-Walcutt et al. 2012). Boredom and fatigue are theoretically differentiated from each other by assuming that boredom is caused by underload, while fatigue is caused by overload (Pan et al. 1994). However, fatigue can be accompanied by decreased arousal (Moore et al. 2017) while boredom can be accompanied by increased arousal (Bailey et al. 1976 cited by Smith 1981). Noting the ambiguity in the literature, we take the perspective of Grandjean (1979 cited by Lal and Craig 2001) that boredom can be considered a special type of fatigue, and thus we will refer to the term “fatigue” within this paper as also including “boredom”.

## Temporal discrimination

Temporal processing in the auditory domain proceeds on multiple time scales in parallel (e.g., Nelken 2004). Temporal discrimination measured with the spatial temporal order judgment (spatial TOJ) task (Fostick and Babkoff 2013; Bernasconi et al. 2011; Szymaszek et al. 2006, 2009; Fink et al. 2005) reveals a temporal window of 20–70 ms duration, below which listeners are not able to reliably judge the order of two consecutive identical tones presented to different ears (Fostick and Babkoff 2013; VanRullen and Koch 2003; Szymaszek et al. 2009; Pöppel 1997, 2004). In the spatial TOJ task, listeners are instructed to judge the order of two short tones delivered to different ears by reporting the ear of the first tone. Compared with the spatial TOJ, the spectral version of this task is likely less sensitive to temporal and more to pattern discrimination (i.e., at short intervals, low–high and high-low pairs can be distinguished by pitch alone, because temporal integration is weighted towards the more recent sound input—see, e.g., Zwislocki 1960), an assumption supported by the finding of short-term learning in the spectral but not in the spatial version of the task (Fostick et al. 2014a; Fostick and Babkoff 2013).

### Continuous performance in the TOJ task

The spatial TOJ threshold has been shown to be sensitive to sleep-deprivation (Fostick et al. 2014b; Babkoff et al. 2005) but not to the time on task (two repetitions of the threshold measurement: Fostick and Babkoff 2013 and Fostick et al. 2014a; three repetitions: Fink et al. 2005). In another study, performance even improved when participants repeatedly judged the order between two tones presented with fixed inter-stimulus interval (ISI) set at their individual threshold level (Bernasconi et al. 2010). This is in stark contrast to the short-term performance decline observed in our previous study (Simon and Winkler 2018). While in Bernasconi and colleagues’ study (2010), feedback was provided after each trial, this was not the case in our previous study. The feedback could have provided additional motivation and/or could have better separated the trials. Furthermore, because in Bernasconi and colleagues’ study (2010), each participant was tested only at one ISI, fine-pattern differentiation learning could have taken place.

### The current study

Our primary goal was to replicate the previously observed performance deterioration and to test whether it can be considered a genuine fatigue effect. Because Simon and Winkler (2018) did not use mandatory pauses between successive TOJ measurements, we will test the above questions by asking whether the task decrement can be eliminated by longer inter-measurement breaks. These breaks will allow task-goals to be replaced by competing goals that can also be pursued in the experimental environment (such as drinking water, mind-wandering, etc.). Based on the goal-conflict approach explanation of fatigue (Hockey 2011), rest periods have the potential to protect against performance decline.

As feedback was the most likely candidate that could explain the differences between our previous results and those of the studies of Bernasconi et al. (2011), we will also test the effects of performance-feedback by providing feedback that compares the participants’ performance to a standard. This procedure is expected to increase the participants’ motivation, as it can be assumed that they do not want to perform below average (Garcia et al. 2006) and thus the need to protect their self-image can strengthen the relative importance of task-performance. In line with both the motivation control theory (Hockey 2011) and the cost–benefit evaluation approach (Boksem and Tops 2008), strengthening the current goal can reduce fatigue effects.

Therefore, we tested the effects of feedback and longer pauses between the repeated TOJ measurements in a 2 × 2 design. It was hypothesized that both of these manipulations can reduce or even eliminate the performance deterioration, because rest and motivation have been shown to be effective factors for reducing mental fatigue (Bills 1931; McCormick et al, 2015; Blasche et al. 2017).

Furthermore, we explored whether there is also a long-term decline in performance or the floor has been reached after only four measurements. To test this putative longer-term effect, the order of different conditions was balanced across participants allowing us to measure the time on task effect on the TOJ thresholds on both shorter and longer time-scales: the 4 threshold measurements within each condition (ca. 5–8 min versus all 16 measurements throughout the whole session (ca. 25–30 min). The presence of a long-term performance decline would appear as larger performance decrement over all 16 than over 4 measurements. Note that this procedure does not accurately assess longer term decline, because participants were allowed to rest between measurements, which could have reset fatigue. On the other hand, finding a long-term decline even under these circumstances would suggest the likely presence of a stronger long-term effect.

Finally, we tested whether the performance decline was due to a general decline in attention or the performance deterioration was specific to processes involved in the TOJ task. To this end an auditory flanker task was completed four times in a row with participants controlling the rest time between successive task blocks, (similar to the TOJ threshold measurement). The flanker task was similar in its makeup (stimuli and procedure) to TOJ task but did not depend on the threshold of temporal discrimination (it differed from the TOJ in having a fixed ISI [100 ms] and in delivering also trials in which both tones presented to the same ear [congruent trials]). Thus while the TOJ and the flanker task share their requirements of executive control processes, only TOJ involves testing the acuity of perceptual processes. Therefore, employing the flanker task allowed us to test whether the performance decline previously observed for TOJ was due to decline in general executive control processes or processes specific to the TOJ task. In the first case, one should expect an increase of the flanker effect (increased reaction time difference between the congruent and incongruent trials) within a period comparable to that found for the TOJ task which correlates with the TOJ threshold increase. Because attentional decline typically becomes detectable only after time-on-task periods which are longer than the duration of our 4 successive TOJ measurements (Faber et al. 2012), we do not expect to find significant increase of the flanker effect.

Three new experiments were run. The first one can be considered as a pilot of the second. However, because it has been completed on a group of reasonable size, thus providing value in terms of replicating the main findings of the second experiment, it is presented, but only in the Supplementary Material (Section A). Here we start with a short description of the relevant incidental finding of our previous study (Simon and Winkler 2018) including details not described in the original report.

## Experiment I (Initial findings)

Simon and Winkler’s study (2018) tested the correlation between the temporal discrimination threshold and perception in the auditory streaming paradigm (Bregman 1990). The first task in the experiment was a TOJ measurement that was followed by different psychometric tasks and questionnaires. Previous reports suggested that the spatial TOJ paradigm has lower test–retest reliability than the spectral version of TOJ (Fink et al. 2005). Therefore, Simon and Winkler study (2018) measured the spatial TOJ threshold again four times in a row after all the other psychometric tasks were completed (approximately 1 h from the beginning of the experiment).

### Methods

All methods are fully described in Simon and Winkler (2018). Here we only repeat those relevant for the current analysis.

### Participants

Forty-one (29 female) healthy young adults participated in the study. They received financial compensation for their participation. All of them had normal hearing, as none of them had a hearing threshold higher than 25 dB HL and the difference between the two ears did not exceed 15 dB HL at the measured frequencies (250, 500, 1000 and 2000 Hz). The average age of the participants was 21.98 (between 18 and 27 years). Participants provided written informed consent to the procedure, which was approved by the institutional Ethical Board (EPKEB). The experiment took place in a sound-attenuated laboratory.

### Stimuli and procedures

#### Temporal Order Judgment task (TOJ)

Participants were instructed to judge the order of two short (10 ms, 1 ms rise/fall times) pure tones (800 Hz) delivered to different ears by reporting whether the sequence started at their right or left ear. The sounds were presented via headphone at 69 dB SPL. The goal of the test is to find the minimal ISI allowing a listener to reliably judge the order of the two tones. Practice was administered in two phases before the threshold measurement reported in the study. In the first phase, feedback was provided after each response (ISI fixed at 150 ms; six repetitions). In the second phase, a summary of the performance was provided after each practice block. Practice blocks consisted of 6 pairs with ISI = 150 ms and 6 pairs with ISI = 100 ms, delivered in a randomized order. There was no time pressure for responding: the next pair was presented 500 ms after the response to the previous pair. During the main threshold measurements, the stimuli were presented 600–900 ms after the response (pressing the “1” or “2” keys on a standard IBM PC keyboard with the index/middle finger for pairs starting at the left or right ear, respectively) to the previous sound pair was registered. The threshold was measured with a three-down-one-up adaptive algorithm that is: after three correct responses, the ISI was shortened by one step; after an error, the ISI was increased by one step. The threshold measurement was stopped after eight errors, and the individual’s threshold was calculated as the average of the last six ISIs at which the individual committed an error. The initial ISI was 120 ms, the initial step size was 20 ms that was halved after each error until it reached the minimum step size of 5 ms. The maximum ISI was 200 ms. One threshold measurement lasted for about 2 min.

#### Procedure

The TOJ threshold was measured five times altogether: first at the beginning of the experimental session and four times after all other measurements. To maintain motivation and attentional focus, the four successive measurements at the end of the session were introduced as a ‘challenge’: a small prize was offered for exceptional performance (less than 15 ms) in the form of a chocolate bar and after each threshold measurement (referred hereafter as “run”) the measured threshold was presented on the screen. Participants were allowed to do the tasks at their own pace: they started the next run by pressing the SPACE key whenever they felt that they are ready for it.

#### Equipment (applicable to all experiments in this study)

All sounds were generated in the Audacity 2.1.1. software (© 1999–2017 Audacity Team), with 44,100 Hz sampling frequency at 16-bit resolution. Sounds were delivered through Creative SB X-Fi sound card and Sennheiser HD 600 headphone by Matlab R2014a software (PsychtoolBox 3.0.12; Brainard 1997; Pelli 1997; Kleiner et al. 2007). Responses were measured by a custom MATLAB script.

#### Questionnaire (PANAS)

The Hungarian version of the Positive and Negative Affect Schedule (PANAS—Gyollai et al. 2011 based on Watson et al. 1988) questionnaire was administered after the first and the last TOJ measurement. The questionnaire presents descriptions of 10 positive and 10 negative emotional states (e.g., excited, vigilant, nervous, vigorous, tense). Participants reported how their actual state matched with each of the 20 items on a 5 point Likert scale (from ‘Not at all’ to ‘Very much’). The state “fatigued” was appended to the states tested by the questionnaire to register a proxy measure of subjective fatigue. The positive affectivity index was calculated as the sum of the response values of the 10 original positive emotional states and similarly the negative emotional affectivity index was based on the responses of the 10 original negative states.

#### Statistical analysis common to all experiments

Spearman’s Rank correlation was applied for all correlational analysis (even when the two variables met all the prerequisites of a parametric probe) for better comparability across the different results (see Supplementary Tables 9 and 10 for a comparison between Spearman’s and Pearson’s correlation results for all correlations tested).

Repeated measures ANOVAs were used to test the effects of the main variables. The Mean Difference (MD), and partial eta square effect size (*pη*^2^) are reported for each analysis. Whenever Mauchly’s Test of Sphericity was significant, the degree of freedom was Greenhouse–Geisser corrected and the *ε* coefficient is reported. The alpha level for statistical tests was set at 0.05. Post-hoc pairwise comparisons were Bonferroni corrected.

### Results (initial findings)

The data are available on the following site: https://osf.io/rauwb/.

**Fig. 1:**
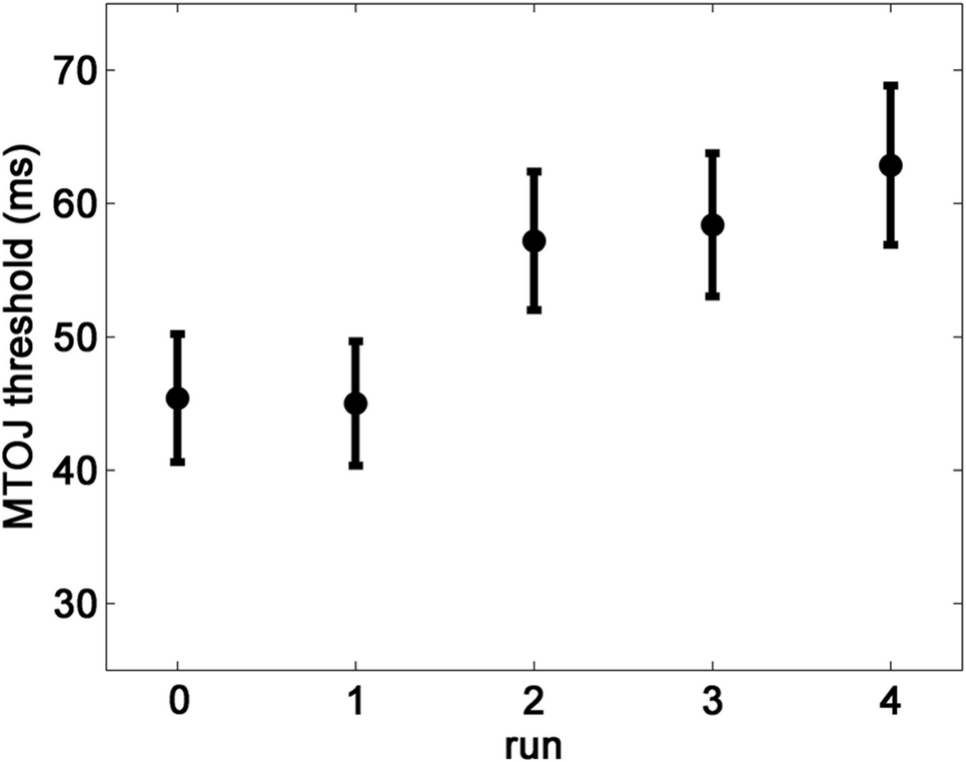
TOJ thresholds from the initial experiment. The first measurement (run = 0) took place at the beginning of the experimental session, whereas the other four were administered without long breaks at the end of the session (ca. 1 h later). Participants received feedback after runs 1–4. The vertical axis shows the average TOJ thresholds with ± 1 SE (standard error)

The average thresholds are shown on Fig. 1. The one-factor rmANOVA of the five TOJ measures was significant [*F*(4,160) = 6.212, *p* < 0.001, *pη*^2^ = 0.134]. Post-hoc comparisons yielded the following significant differences: between runs number 0 and 4 (MD = − 17.48, *p* = 0.001), between runs number 1 and 2 (MD = − 12.19, *p* = 0.04), and between runs number 1 and 4 (MD = − 17.87, *p* = 0.004).

The change in subjective fatigue was also significant (*t*(39) = − 4.207, *p* < 0.001).

#### Correlational analyzes

Significant correlation was found between the change (end-minus-beginning) in subjective fatigue (measured together with PANAS) and the change in positive affectivity [*r*_s_(39) = − 0.404, *p* = 0.016] but not between the change in subjective fatigue and the change in negative affectivity [*r*_s_(39) = 0.192, *p* = 0.236].

No significant correlation was found between performance deterioration in TOJ (threshold at the 4th minus the 1st measurement) and subjective fatigue either at the beginning [*r*_s_(39) = 0.128, *p* = 0.426] or at the end of the session [*r*_s_(38) = − 0.053, *p* = 0.746] or the change in subjective fatigue during the session [*r*_s_(38) = − 0.066, *p* = 0.688].

### Discussion (initial findings)

There was a significant increase of the threshold during the four consecutive measurements, even though one measurement lasted only for ca. 1–2 min, there was no time-pressure for the responses, and participants themselves determined when to start the next measurement (i.e., the inter-measurement interval). On the other hand, there was no significant difference between the threshold found during the first TOJ measurement at the beginning of the experimental session and the first of the last four TOJ thresholds measured at the end of the experimental session. This suggests that general increase in sleepiness or task-disengagement did not significantly influence the participants’ performance in the threshold measurement between the beginning and the end of the session. Therefore, three main non-exclusive explanations can be offered: (1) The TOJ task requires some perceptual process (such as the subtle discrimination required), which cannot be maintained without resting or strong effort; (2) Performance deterioration is the result of decline in control mechanisms (e.g., attention). (3) Participants commitment to do the task properly (thoroughly as opposed to formally following the instructions) decreased during the successive measurements.

## Experiment II

In a pilot experiment, we replicated the short-term performance deterioration of the TOJ threshold (see Suppl. Section A). This motivated us to conduct a full study with a relatively large sample size. If the short-term increase of the TOJ threshold is a fatigue effect one should expect that proper rest periods and strong motivation would attenuate or eliminate it. Therefore, we employed two types of manipulations in a 2 × 2 design: mandatory resting periods between consecutive measurements and a false feedback comparing the participant’s actual threshold to a “standard”. The latter was expected to provide stronger motivation than only presenting the actually measured threshold value on the computer screen as it was done by Simon and Winkler (2018). The effects of the valence of the initial feedback (positive or negative) were also explored.

Because performance deterioration can be the result of fatigued control mechanisms without change in temporal processing, an auditory flanker task was also administered to assess attentional control decline falling outside changes in the resolution of temporal processing. The flanker task administered contained stimulus pairs with a long ISI that allowed participants to make the necessary order judgements without too much effort focused on the perceptual aspect of the task (Pöppel 1997). This task was repeated four times (just as the TOJ measurements) with 40 incongruent trials (when the sounds were presented to different ears, just like in TOJ) per run. The number of incongruent trials in the flanker task is roughly equal to the number of judgements made during a TOJ threshold measurement. Also similar to the TOJ threshold measurements, each stimulus block was initiated by the participant. Performance change from run to run in the TOJ task should then be corrected with the changes observed in this task for assessing the role of fine temporal discrimination in performance decline in TOJ.

Another possible explanation to the performance decline is change in the participants’ willingness to properly perform the task (i.e., they could abandon the task and respond randomly). To test this, we measured the strength of task-goals (Elliot et al. 2011). This questionnaire measures the willingness to comply with task demands by presenting items, such as ‘Do the task correctly’ or ‘Avoid doing the task incorrectly’. The questionnaire was validated on undergraduate students completing an exam. If performance deterioration was due to reduced willingness to perform the task at a high level, then the task-goals score should reflect this.

Furthermore, if a putative motivation reduction in the previous study was at least partly due to how participants evaluated the difference between the stated goals (15 ms) and their performance, then participants more sensitive to criticism would show a larger performance deterioration with the current procedure (which directly, although falsely, relates their performance to an expected level), because they either get more frustrated during the tasks or less motivated to continue. On the other hand, the rationale for linking the subjective feeling of fatigue and sensitivity to criticism is that participants more sensitive to the negative evaluation of their performance could be more willing to report being fatigued as failure can be attributed to something unrelated to their abilities. Note that this suggests association between sensitivity to criticism and subjective, rather than objective fatigue. To test this possibility, we measured sensitivity to criticism by a new questionnaire (personal communication by Gábor Orosz). This construct measures the negative emotional impact of a received critique that manifests either in over-engagement with the critique or in the effort to suppress critique-related thoughts.

Finally we also assessed the relationship between the subjective and the objective indices of fatigue. Because some previous studies showed that the two constructs are uncorrelated under non-extreme conditions while others found correlation (see Sect. 1.1), here we assumed that subjective fatigue will be better explained by the sensitivity to criticism than by change in performance (objective fatigue).

### Methods

#### Participants

Forty-eight plus one healthy, native Hungarian speaking young adults participated in the experiment. They received a modest financial compensation (1410 HUF, ~ 5 USD) for their participation through a student work organization. One participant reported that she heard the sounds louder in her left ear (despite normal audiometry), therefore she was excluded and another participant was recorded with the design parameters (see below) assigned to her. In the final group of 48 participants, there were 33 females; the age of participants ranged between 18 and 27. All participants had normal hearing with a hearing threshold below 25 dB HL at 1000 Hz (mean = 4.58; range − 5 to 25) and the difference between the ears did not exceed 15 dB HL (mean = 0.416; range − 15 to 20). Participants provided written informed consent to the procedures, which were approved by the Ethical Board (EPKEB).

#### Stimuli and procedures

*TOJ task*. The same algorithm was used as was employed by Simon and Winkler (2018). The average number of responses per participant was 37.89 (SD = 4.93; minimum = 19.81, maximum = 44). One run lasted typically less than 2 min (most frequently just about 1 min).

In two conditions, mandatory pauses were inserted between runs: for one minute classical music was played and a natural scene was presented on the monitor in front of the participant, which has been shown to have good restorative effect (Berto 2005). The music was selected on the basis of the ratings from 12 young adults (none of whom participated later in the experiment). Three samples out of 12 were selected for the study, the three most neutral (details in Suppl. Section G).

In two conditions, feedback was provided after each threshold measurement. In the mandatory pause + feedback condition, the feedback followed the rest period. Thus the four conditions were: F-P (condition with feedback and mandatory pauses between the measurements), F-NP (condition with feedback but no mandatory pause), NF-P (condition without feedback but with mandatory pauses) and NF-NP (a condition without feedback or mandatory pauses). The feedback was false, one variant being positive, the other negative.

Positive: Congratulations! You performed better than the average of young adults.

Negative: Regrettably, you performed worse than the average of young adults.

Two groups of participants were formed. The experimental procedure for the groups only differed in the feedback they received. Within the two blocks (conditions) with feedback, one group received positive feedback after the first run (measurement; “positive start” group), while the other group received negative feedback first (“negative start” group). Both groups then received negative feedback after the second and third run and positive feedback after the fourth run.

Participants were informed that accuracy is more important than speed in making their judgments.

*Situational Subjective Fatigue Questionnaire* The participants were asked to answer on a 7 point Likert scale (1—‘not at all true’, 7— ‘absolutely true’) to the Hungarian version of the following questions: ‘I felt like my brain got tired during the tasks.’, ‘I frequently felt exhausted during the tasks.’, ‘I felt like I lost efficacy with time on task.’, ‘I became indifferent regarding my performance.’, ‘Sometimes I felt like I want to quit trying.’, ‘It became harder to focus on the task’. As responses to the second item did not correlate with those to the other items, the average of the remaining five items was treated as a proxy of task-related fatigue. The Cronbach’s Alpha of the five-item questionnaire was 0.836.

*Achievement Goals Questionnaire (AGQ)* This questionnaire has been adapted to Hungarian by Urban et al. (2014) from Elliot et al. (2011) original measure. It consists of 18 items evaluated on a 7 point Likert Scale (e.g., ‘My goal was to perform well in the task.’; response alternatives: 1—‘Not true in my case’ to 7—‘Excessively true in my case’). The following subscale scores can be computed: task-approach, task-avoidance (in the sense that someone wanted to avoid failure in the task), other-approach, other-avoidance, self-approach and self-avoidance. In this study, we focused on the first four subscales to assess consistency in the motivation to perform well in the different tasks (task-goals) and to test the effects of our manipulations (other-goals). AGQ was measured two times: once after all TOJ measurements and once after the flanker task.

*Sensitivity to criticism*. As no instrument was readily available to measure one’s criticism-related engagement, we opted to administer a short questionnaire that has recently been constructed by Orosz et al. (in preparation) and validated on > 900 Hungarian responders (from adolescence to elderly age). For details see Suppl. Section F. In the current study, we only analyzed items of the over-engagement and disengagement subscales and pooled them as a proxy to sensitivity to criticism, because the two subscales are positively correlated and they both represent negative aspects of sensitivity. Over-engagement refers to being overtaken by negative emotions (such as worry), ruminating on the criticism and being judgmental of oneself after a negative feedback (e.g., ‘I’m not forgiving with myself, because I constantly think about what was said to me’). Disengagement is also a maladaptive form of emotion regulation and it manifests in denial, distraction, or suppression of the critique-related thoughts (e.g., ‘I do my best to eliminate the pain of the criticism’). The six-item version has an acceptable level 0.804 Cronbach’s Alpha.

*Auditory Flanker Task *This task was based on the auditory attention paradigm of Spagna et al. (2015) but modified to better fit the current experimental setup: instead of frequency we used spatial congruence vs. incongruence to better match the TOJ paradigm. Spagna et al.’s paradigm represents a three-component model of attention: orientation, alerting and executive attention. We focused on the executive component of this attention model, because we assumed that this can be most susceptive to short-term fatigue and it was reported that the measures of the other attention components had low reliability (Spagna et al. 2015). Therefore, orienting and alerting cues were not used and the overall measurement became shorter. Executive attention is reflected in the performance difference between the congruent and incongruent trials. Participants were instructed to judge which of their ears received the first of two successive sounds. In congruent trials, the two sounds were presented to the same ear; in the incongruent trials, the two were presented to separate ears. The sounds and the response buttons were the same as in the TOJ paradigm. The inter-stimulus interval was always 100 ms between the two tones. Congruent and incongruent trials were equiprobably intermixed. The participants performed four runs of this task, each containing 40 congruent and 40 incongruent trials. Similarly to the TOJ measurements, participants started the next run by hitting the SPACE key when they were ready to continue. A practice session preceded the main measurements, 20 repetitions with feedback (at the end of the 20 trials a message about the hit and miss rate appeared in the center of the screen) was provided.

#### Procedure

Participants were assigned to the two groups randomly but with overall equal number of participants being assigned to each. The session started with the audiometry measurement. Participants then completed the practice version of the TOJ task followed by the four blocks of TOJ task (each block containing four runs). The order of the blocks (conditions) was balanced across participants so that each possible order was assigned to exactly one participant in each group.

The TOJ measurements were followed by the computerized version of the questionnaires in the following order: Situational Subjective Fatigue, Achievement Goals, Mindfulness (see Suppl. Section D) and Subjective Vitality and General Subjective Fatigue (see Suppl. Section D)*.* After the questionnaires, participants completed four runs of the Auditory Flanker Task followed by a second administration of the Achievement Goals Questionnaire. The experimental session lasted about one and a half hour (including rests and information at the beginning), taking place on a single day. The order of the TOJ and Flanker sessions was not counterbalanced because that would have introduced noise in the correlation between the potential performance declines measured in the two tasks. With the flanker task performed close to the end of the session, it was assumed that if there is a general increase in fatigue by time-on-task, there is a higher chance that it will manifest in it.

### Statistical principles

The experimental questions were tested with mixed ANOVAs. For assessing the effect of time on task within a measurement block, all four measurements have been included in the ANOVA (factor termed RUN1-4). For testing the manipulation effects, the first runs of the blocks were excluded as those were not preceded by feedback or controlled pauses (factor name: RUN2-4). The long-term performance decline was tested on all 16 consecutive TOJ measurements irrespective of the manipulations, as the order of those was counterbalanced across participants. Details of the statistical and power analyzes can be found in Suppl. Section E.

Normality was tested with the Kolmogorov–Smirnov tests as the sample size is relatively high. The Feedback-effect and the additional Pause-effect became significant (*p* = 0.018, and *p* = 0.027 respectively); therefore Spearman’s rank correlations were conducted.

### Results

The data are available on the following site: https://osf.io/7832b/.

#### Analysis of TOJ effects

**Fig. 2:**
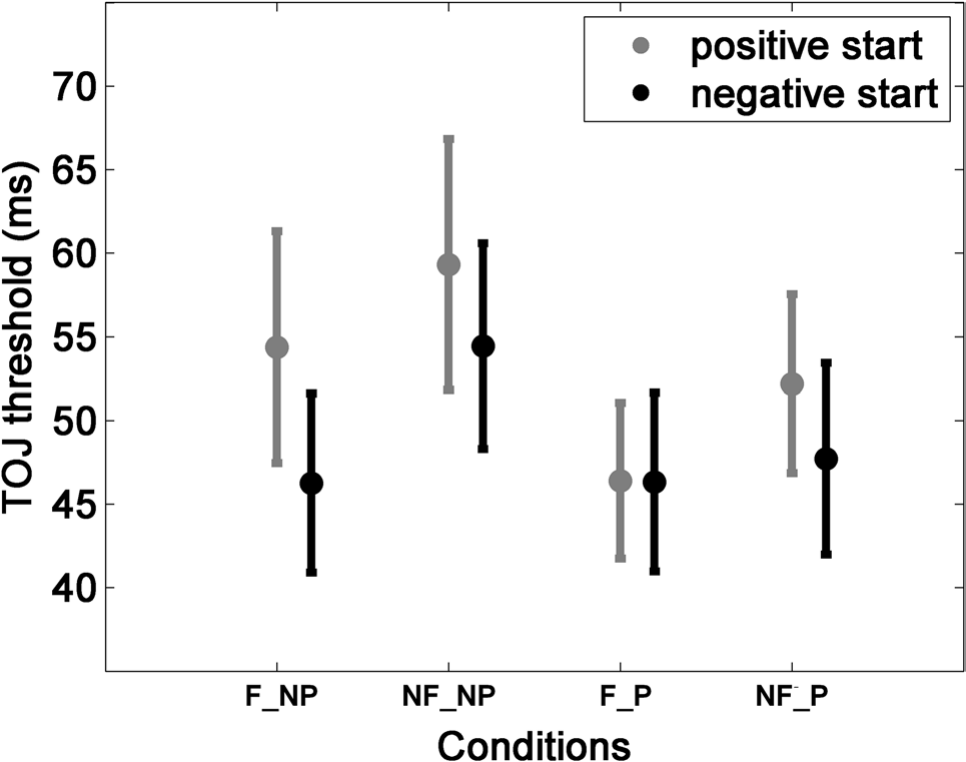
The mean TOJ thresholds by group and by condition with 1 standard error. (*F* feedback, *P* pause)

The mean TOJ thresholds in milliseconds in the “positive start” group were the following: 46.39 (F-P), 52.19 (NF-P), 54.37 (F-NP) and 59.32 (NF-NP). These averages were the following in the “negative start” group: 46.30 (F-P), 47.70 (NF-P), 46.24 (F-NP) and 54.42 (NF-NP)—see also Fig. 2.

TOJ performance as a function of the run was tested by a mixed-model ANOVA (within-subject factor RUN1-4 × between-subject factor GROUP [positive vs. negative first feedback]) of the TOJ thresholds measured in “no pause, no feedback” condition [which was assumed to be compatible with the measurement in Simon and Winkler (2018)]: significant main effect of RUN1-4 (*F*(3,138) = 3.420, *p* = 0.019, *pη*^2^ = 0.069) and significant RUN1-4 × GROUP interaction [*F*(3,138) = 3.309, *p* = 0.022, *pη*^2^ = 0.067] were found. While there was significant performance deterioration in the “positive start” group [*F*(3,51.053) = 4.007, *p* = 0.021, *ε* = 0.740], only a tendency for deterioration was observed in the “negative start” group [*F*(3,69) = 2.705, *p* = 0.052]. Neither covariate from the flanker task affected the TOJ threshold deterioration significantly: (a) change from the fourth compared to the first run in error rate difference between the congruent and incongruent trials [*F*(2.59,116.56) = 0.552, *p* = 0.622, *pη*^2^ = 0.012] and (b) mean RT difference between the congruent and incongruent trials [*F*(3,135) = 0.773, *p* = 0.511, *pη*^2^ = 0.017].

There was no significant difference between the two groups in the first (pre-manipulation) threshold [*t*(46) = − 0.374, *p* = 0.710, M(positive) = 44.06 ms, M(negative) = 47.05 ms].

The four-way mixed-model ANOVA (within-subject factors PAUSE × FEEDBACK × RUN2-4 × between-subjects factor GROUP) testing the effects of the manipulations yielded significant main effects of PAUSE [*F*(1,46) = 4.784, *p* = 0.034, *pη*^2^ = 0.094] and FEEDBACK [*F*(1,46) = 8.946, *p* = 0.004, *pη*^2^ = 0.163] and a significant GROUP × RUN2-4 interaction [*F*(2,92) = 0.4.493, *p* = 0.014, *pη*^2^ = 0.170]. Both feedback and pause reduced the thresholds (see also Suppl. Figure 8). Post-hoc analyses revealed that whereas in the “positive start” group there was a significant RUN effect [*F*(2,46) = 5.571, *p* = 0.007, *pη*^2^ = 0.195], this was absent in the “negative start” group [*F*(2,46) = 0.603, *p* = 0.552, *pη*^2^ = 0.026].

The mean pause effect (collapsed across groups and runs 2–4) was 6.92 ms (NF-NP minus NF-P), the feedback effect was 6.56 ms (NF-NP minus F-NP), the additional mean pause effect (the pause effect when feedback was provided) was 3.95 ms (F-NP minus F-P) and the additional mean feedback effect (the feedback effect when pause was mandatory) was 3.59 ms (NF-P minus F-P). Correlations between these differences are summarized in Table 1 (see Pearson’s correlations in Supplementary Table 10).

**Table 1:** Rho correlation coefficients between the manipulation effects

	Feedback	Add. pause	Add. feedback
Pause	**0.357** (*p* = 0.013)	**0.434** (*p* = 0.002)	**− 0.374** (*p* = 0.009)
Feedback		− 0.218 (*p* > 0.05)	−0.155 (*p* > 0.05)
Add. pause			**0.309** (*p* = 0.033)

The abbreviation ‘Add.’ (additional) refers to the feedback effect when also a pause was mandatory and to the pause effect when also feedback was provided

Bold values indicate statistically significant correlations

**Fig. 3:**
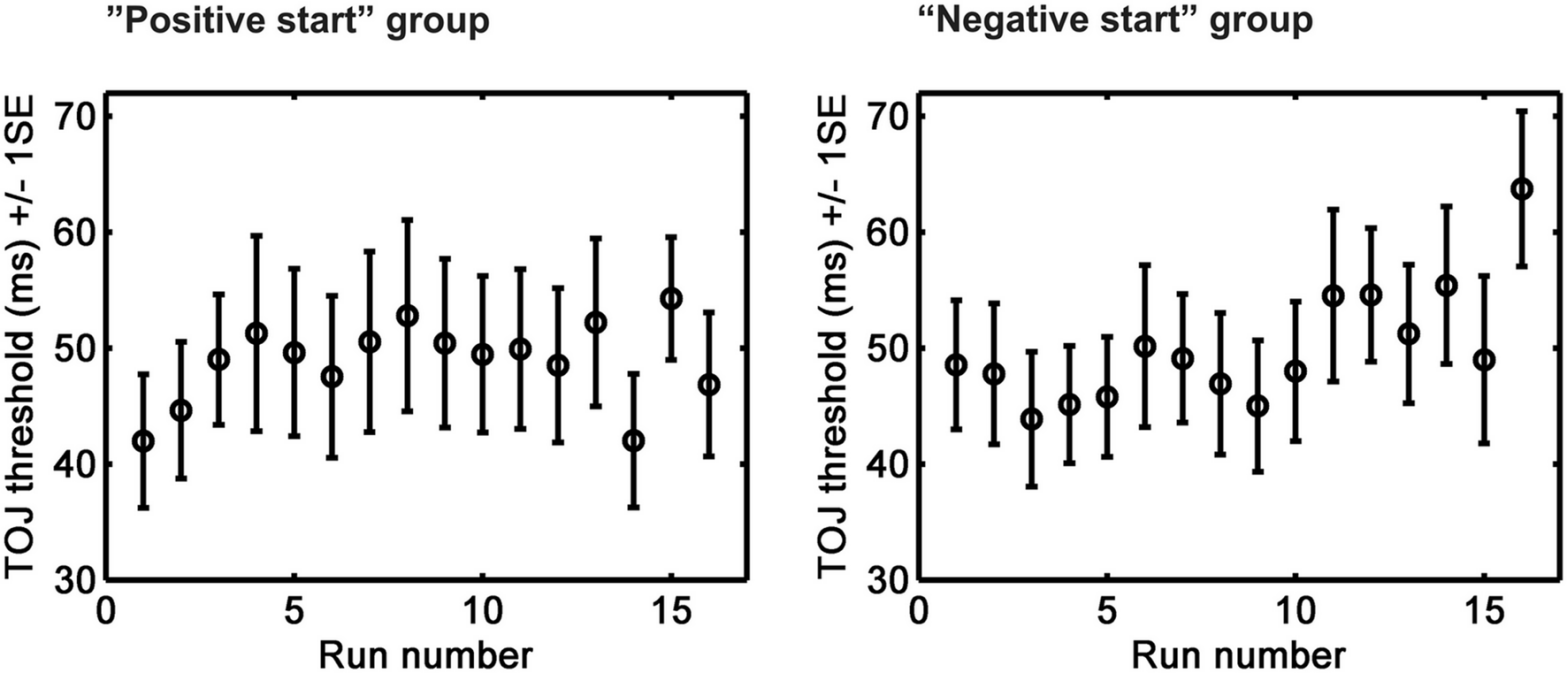
Group-average (*N* = 24) TOJ thresholds (with ± 1 SE) for the 16 consecutive measurements

The ANOVA (within-subject factor TIME [*N* = 16] × between-subjects factor GROUP) testing TOJ performance change throughout the whole series of measurements (all 16 runs as a sequence) yielded significant interaction between TIME and GROUP [*F*(15,690) = 2.082, *p* = 0.009, *pη*^2^ = 0.043]. Figure 3 shows that the performance differed between the two groups as a function of the position of the threshold measurement within the whole series. Performance in the “positive start” group dropped early and stabilized thereafter, whereas in the “negative start” group, performance started to deteriorate only at the end of the series of measurements.

Finally, the mixed ANOVA (within-subject factor ORDER [the first vs. the second run that included feedback] × between-subject factor GROUP) testing the immediate effects of the valence of the first feedback yielded no significant main effect or interaction.

The mixed-model ANOVA of the AGQ scores (within-subject factors TYPE [Task-goals vs. Other-goals] × QUALITY [approach vs. avoidance] × REPORT-TIME [post-TOJ vs. post-flanker task] × between-subjects factor GROUP) yielded a significant TYPE effect, task-goals scores being higher than the other-goals scores (*F*(46) = 34.58, *p* < 0.001, *pη*^2^ = 0.429). The TYPE × QUALITY interaction was significant [*F*(46) = 14.468, *p* < 0.001, *pη*^2^ = 0.239; stronger avoidance in other-goals, stronger approach in task-goals] as well as the REPORT-TIME × QUALITY interaction [*F*(46) = 4.88, *p* = 0.032, *pη*^2^ = 0.096; decreased avoidance goals after the flanker than the TOJ task; see also Suppl., Section C, Fig. 6]. No other main effect or interaction was significant. The mean scores of task-goals (approach and avoidance combined) were 5.81 and 5.75 in the post-TOJ and post-flanker measurements, respectively, while the mean scores of other-goals were 4.67 and 4.35, similarly.

#### The flanker task

The ANOVA (within-subject factors RUN1-4 × CONGRUENCY [congruent vs. incongruent trials] × between subject factor GROUP) of the median reaction times showed a significant CONGRUENCY main effect [*F*(1,46) = 69.239, *p* < 0.001, *pη*^2^ = 0.601], but no RUN1-4 effect [*F*(3,138) = 0.109, *p* = 0.95, *pη*^2^ = 0.002] and no significant interactions. The mean incongruent-minus-congruent differences in the four consecutive runs are shown in Suppl. Table 8. The ANOVA of error rates yielded significant RUN1-4 [*F*(3,138) = 4.16, *p* = 0.007, *pη*^2^ = 0.083] and CONGRUENCY main effects [*F*(1,46) = 63.241, *p* < 0.001, *pη*^2^ = 0.579] and RUN1-4 × GROUP interaction [*F*(3,138) = 3.141, *p* = 0.027, *pη*^2^ = 0.064]. The mean error rate differences are shown in Suppl. Table 8, Section C. The effect of the TOJ threshold covariate on the CONGRUENCY [*F*(1,45) = 31.850, *p* < 0.001, *pη*^2^ = 0.414] as well as on the RUN factor [*F*(3,135) = 2.847, *p* = 0.040, *pη*^2^ = 0.059] were significant, showing that the TOJ threshold had a significant role in determining the error rates.

#### Analysis of individual differences

The subjective situational fatigue showed stronger relationship with the “sensitivity to criticism” score [*r*_s_(46) = 0.45, *p* = 0.001] than with TOJ performance deterioration (objective fatigue: change from the first to the fourth run of the NF-NP condition), the latter not reaching significance [*r*_s_(46) = − 0.21, *p* = 0.15]. No significant correlations were found between TOJ performance deterioration and any of the questionnaire measures or the change from the first to the fourth run in the flanker task performance (congruent-minus-incongruent RT).

## Experiment III

The order of the TOJ and the Flanker task was not counterbalanced in Experiment II, which could have biased some of the Flanker task results by, e.g., carryover from the TOJ task or by knowing that the Flanker is the last auditory task of the session. Therefore, in Experiment III, the order between the TOJ and the control task was counterbalanced. Since we did not find a time-on-task effect on the flanker effect (the congruent-incongruent difference), we modified the task to be even more similar to a TOJ measurement by removing the congruent trials. Thus the task only differs from the TOJ task by having a constant 150 ms ISI (labeled Easy-TOJ). The ISI was increased compared to that in the previous experiment, because in Experiment II, some participants’ first TOJ threshold in the NF-NP condition was higher than 100 ms (but not higher than 150 ms). The benefit of the change is that the duration of one block of the modified task is equal to that of the TOJ threshold measurement. Finally, six consecutive blocks were presented for each task, instead of the four used in previous experiments to assess whether the trend of performance decline continues beyond four consecutive blocks. Feedback was only provided at the end of the experimental session for assessing performance decline effects without the feedback effect found in Experiment II.

### Methods

#### Participants

Two groups of twenty healthy, native Hungarian speaking young adults participated in the experiment: The first group included 18 females (70% right handed; age between 18 and 23 years), the second 18 females (95% right handed; age between 18 and 28 years). All participants had normal hearing with a maximum 20 dB HL hearing threshold at 1000 Hz (mean = 4.625 dB; range − 5 to 20) and the difference between the ears did not exceed 10 dB HL (mean = − 1.75 dB; range − 10 to 10). Participants provided written informed consent and received course credit as compensation for their participation. The study was approved by the Ethical Board (EPKEB).

#### Stimuli and procedures

The TOJ task was identical to that employed in Experiment III. The Easy-TOJ task was a reduced version of the previously used flanker task: there congruent trials were removed and the constant ISI was set at 150 ms. This task thus only differs from the TOJ task in that ISI is fixed. One stimulus block included 40 trials.

*Task Specific Fatigue Question* Participants were asked to rate with a 7 point Likert scale (1—Not at all, 2—No, 3—Rather not, 4—Yes and No, 5—Rather true, 6—True, 7—Absolutely true) how much they agree with the following statement: “I felt like my brain got tired during the tasks”.

*Task Specific Effort Question* Participants answered how much effort they needed in the previous task using a 9 point Likert scale (1—Nothing, 9—Very much).

*Task Specific Difficulty Question* Participants answered using a 9 point Likert scale (1—Nothing, 9—Very much) how difficult the previous task was.

*PANAS* The affectivity questionnaire presented in Experiment I with the change that the expression “bored” was inserted at the 11th position of the list (but not used when calculating either of the PANAS affectivity indices).

*AGQ* Same as in Experiment II.

*Procedure* Participants were briefed at the beginning of the experiment that performance feedback will be presented only at the end of the experiment (except for practice blocks) in terms of their average accuracy and average discrimination threshold. The main experiment consisted of two parts separated by a 3-minute mandatory pause during which instrumental music was playing in the background. Listening to music was optional: participants were told that if they do not like the music, they can take off the headphone. In each part, there were either six stimulus blocks of the same type: half of the participants started with the TOJ threshold measurements (1st/TOJ-start group) while the other half with the Easy-TOJ task (2nd/Easy-TOJ-start group). The practice sessions were identical to those employed in Experiment II, with a separate practice presented before the first block in each part of the experiment. These measurements were preceded and followed by the PANAS Questionnaire. Before the post-measurement PANAS, the questions about effort and perceived task difficulty were presented. The PANAS was followed by the Task Specific Fatigue question. Finally, the AGQ questionnaire was to be filled with its questions referring to all the tasks before.

### Statistical testing

The same principles were applied as in the previous experiments. As an index of performance decrement, a linear function was fitted to the six measurements and its slope was calculated (termed TOJ-trend and Easy-TOJ-trend).

### Results

The data are available on the following site: https://osf.io/c2hbv/.

#### Descriptive statistics

The average TOJ threshold was 38.45 ms (SD = 26.54 ms) in the first block and 51.22 ms (SD = 22.86 ms) for all six blocks. The average accuracy was 97.25% (SD = 3.4%, minimum 88%, maximum 100%) in the first block of the Easy-TOJ task and 96.15% (SD = 4.17%, minimum 83%, maximum 100%) for all six blocks. The average task-approach goal value was 5.74 out of 7 (SD = 1.33; the other goals are reported in the Supp. Table 14–15), which can be regarded as rather high, as it is significantly greater than four that is ‘moderately true’ [*t*(39) = 8.229, *p* < 0.001]. The mean response to the task-specific fatigue question after the TOJ measurements was 4.1 (SD = 1.53), which suggests the presence of subjective fatigue, because this is significantly [*t*(39) = 4.53, *p* < 0.001] higher than 3 (‘rather no’), which is the highest no-fatigue response.

#### Analysis of the time on task effect on the TOJ threshold

**Fig. 4:**
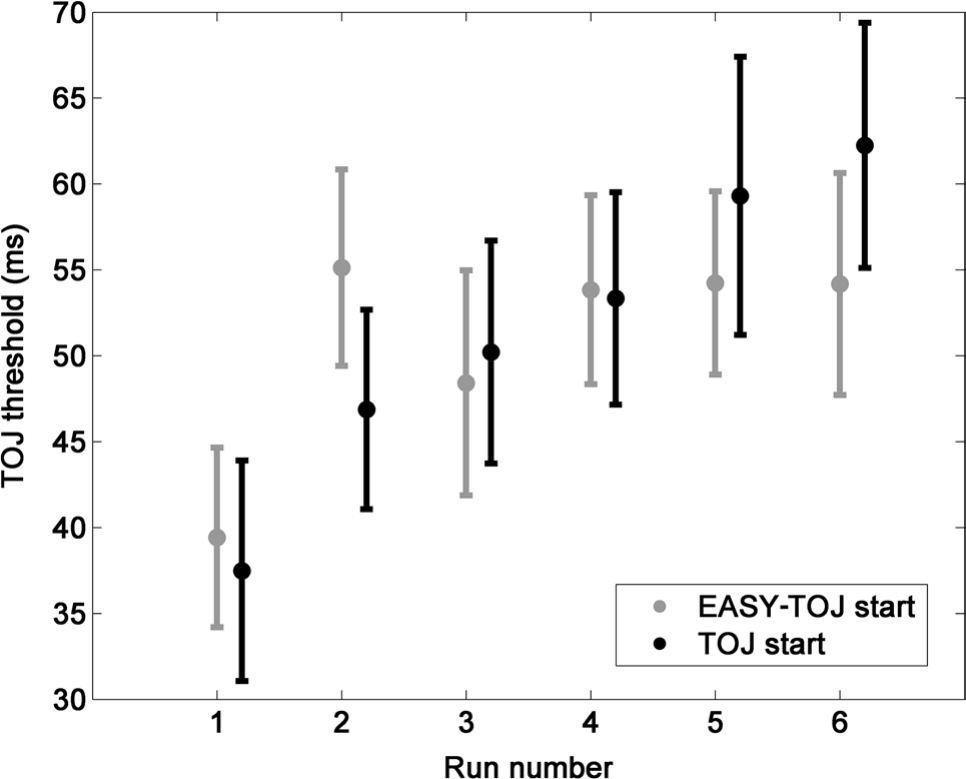
Performance deterioration during successive TOJ threshold measurements, separately for the two groups of subjects. The error bar reflects the standard error

Figure 4 shows the TOJ thresholds as a function of the order of measurements (RUNs), separately for the two groups of participants. An ANCOVA was conducted on the TOJ threshold with the within-subject factor RUN (*N* = 6), the between-subject factor GROUP (*N* = 2; TOJ start vs. Easy-TOJ start), and the Easy-TOJ-trend as covariant. There was a significant main effect of RUN [*F*(5,185) = 4.774, *p* < 0.001, *pη*^2^ = 0.114], but no significant GROUP effect [*F*(5,185) = 0.943, *p* = 0.943, *pη*^2^ = 0.025] or Easy-TOJ-trend effect [*F*(5,185) = 0.686, *p* = 0.635, *pη*^2^ = 0.018]. Exchanging the covariant to the mean Easy-TOJ accuracy resulted in, the RUN effect no longer being significant [F(5,185) = 0.1.37, *p* = 0.238, *pη*^2^ = 0.036]. However, the covariant still had no significant effect [*F*(5,185) = 1.23, *p* = 0.296, *pη*^2^ = 0.032].

### Analysis of the time on task effect on the Easy-TOJ accuracy measurement

Accuracy as a function of the order of measurements (RUNs) is shown on Supplementary Fig. 9. An ANCOVA was conducted on the Easy-TOJ accuracy with the within-subject factor RUN (*N* = 6), the between-subject factor GROUP (*N* = 2; TOJ start or Easy-TOJ start), and the covariant TOJ-trend (to assess the commonality with the slope of accuracy in the Easy-TOJ task). There was a significant main effect of RUN [*F*(3.88,143.73) = 4.089, *p* = 0.004, *pη*^2^ = 0.100, *ε* = 0.777], but no significant GROUP effect [*F*(3.88,143.73) = 1.177, *p* = 0.324, *pη*^2^ = 0.031, *ε*  = 0.777] or TOJ-trend effect [*F*(3.88,143.73) = 0.315, *p* = 0.863, *pη*^2^ = 0.008, *ε*  = 0.777]. Exchanging the covariant to the mean TOJ threshold (to check whether the effects were dependent on the temporal processing parameter represented by the TOJ threshold), the RUN effect disappeared [*F*(3.73,138.34) = 0.821, *p* = 0.536, *pη*^2^ = 0.100, *ε* = 0.748] and the covariant effect became significant [*F*(3.73,138.34) = 3.692, *p* = 0.008, *pη*^2^ = 0.091, *ε* = 0.748].

#### Correlations

There was a significant correlation between the two measures of subjective fatigue: the PANAS post-TOJ fatigue question and the task-specific fatigue question after TOJ measurements [*r*(38) = 0.488, *p* = 0.001]. We checked the correlations of the latter with other variables as it is more specific to the TOJ task (to assess the commonality with the slope of accuracy in the TOJ task). There was a significant correlation between the change in positive affectivity and subjective fatigue [*r*(38) = − 0.346, *p* = 0.029], larger decrease in positive affectivity was related to higher subjective fatigue. However, there was no significant correlation between the TOJ-trend (a proxy of objective fatigue) and subjective fatigue [*r*(38) = − 0.114, *p* = 0.483].

The correlation between mean TOJ threshold and mean Easy-TOJ accuracy is rho(38) = − 0.719, *p* < 0.001). For a full list of correlations, see Supp. Table 12.

#### Predictors of subjective fatigue

To explain the subjective fatigue measure a linear regression was conducted using the backward elimination method. The independent variables were: perceived effort, general perceived fatigue before the TOJ tasks, change in positive affectivity and the minimum TOJ threshold. These variables were selected because they had a correlation higher than 0.3 with the dependent variable and did not have a correlation higher than 0.7 with each other—due to the latter, perceived effort was included, while task difficulty was not used in this analysis (see Supp. Section H after Supp. Table 13). The model was significant [*F*(3,35) = 7.137, *p* < 0.001] and had an adjusted R Square of 0.386. Based on the standardized coefficient beta [*β* = 0.347, *t*(35) = 2.70, *p* = 0.01] and the semi-partial correlation reflecting the unique contribution of the variables (part = 0.340) the most predictive variable was perceived effort, while the second most predictive one was the minimum TOJ threshold (*β* = 0.325, *t*(35) = 2.54, *p* = 0.016; part = 0.319). Before-task fatigue (*β* = 0.260, *t*(35) = 1.99, *p* = 0.054; part = 0.319) and change in positive affectivity (*β* = − 0.221, *t*(35) = − 1.69, *p* = 0.099; part = − 0.212) did not significantly predict subjective fatigue themselves, but they improved the adjusted square and were thus kept in the model. Note that the sample size is small.

Further analyses can be found in the Supplement (Section H).

## Discussion

The short-term performance deterioration while repeatedly measuring the spatial TOJ threshold initially observed by Simon and Winkler (2018) has been now replicated multiple times in different groups of participants (including the pilot experiment—see Suppl. Section A). Therefore, it appears to be a reliable phenomenon.

This performance deterioration was eliminated or at least largely reduced by mandatory pauses inserted between successive measurements as well as by a (false) feedback procedure, which strongly motivated the participants to perform the task to the best of their abilities. Again, the effects of pause and feedback have been replicated in two groups of participants (including the pilot experiment—see Suppl. Section A). The effectivity of additional motivation and rest periods is in line with the notion that the deterioration of task performance was due to some form of fatigue (Bills 1931; McCormick et al. 2015; Blasche et al. 2017).

An alternative to the fatigue explanation is that the commitment of participants to thoroughly follow the instructions has diminished during the repeated measurements. In both the main and the pilot experiment (see Suppl. Section A), the willingness of the participants to perform the task was high, ca. 6 out of 7 in the Achievement Goal Questionnaire even after the TOJ measurement. Therefore, it is unlikely that the quick performance deterioration was due to a reluctance to follow instructions. Further, sensitivity to criticism was not related to performance deterioration. This result also argues against a decrement in the willingness to perform the task.

Two non-exclusive versions of the fatigue explanation were offered: fatigue specific to the temporal processing required by the threshold judgments in TOJ and a general increase of inattentiveness. The lack of performance decline in the flanker task (no significant change in the congruent-minus-incongruent RT difference as a function of the run) and the lack of covariate effect of the flanker-task performance on the TOJ performance decline suggests that temporal processing was affected. Note that the error rate increase could be explained by the level of temporal discrimination ability of the participants, as the mean TOJ threshold explained considerable variance in Flanker error rate. However, performance increase is possible when participants know that they reach the end of work causing mental fatigue. This may have affected the performance measured in the flanker task. To asses this putative effect, Experiment III counterbalanced the order of the TOJ and a control task, the latter measuring the accuracy of order judgements at a supra-threshold ISI. The order of the two tasks did not significantly affect performance in either task or the time-on-task effects found for them. Both the TOJ threshold and the Easy-TOJ accuracy showed significant RUN effects, which were eliminated by including the mean value of the other as covariant. This suggests a common source for the two RUN effects. Because only the mean TOJ threshold had a significant covariant effect, the common source is the TOJ threshold, similar to the source of the error increase in the Flanker task in Experiment II. This interpretation is further supported by the significant negative correlation between mean TOJ threshold and the mean Easy-TOJ accuracy, which shows that participants with a high TOJ threshold (low temporal resolution) were less accurate in the control tasks based on order judgement (Flanker and Easy-TOJ tasks) and with the time-on-task increase of the TOJ threshold, they made even more errors in the control tasks. Therefore, we conclude that the short-term TOJ threshold increase is likely due to fatigue-induced changes in fine temporal processing. However, this does not mean that attentional processes were unaffected. During continuous performance, small lapses of attention can happen that can be compensated in other tasks and do not show on a block level average (Hockey 2011). However, the adaptive procedure employed for the TOJ threshold measurement may be more sensitive to these small lapses.

Future research may further explore whether temporal processing per se is highly sensitive to fatigue within such a relatively short period of time or some process specific to the applied TOJ task causes the performance deterioration. For example, in the current study participants had to prepare for the next trial immediately after a response. If they initiated each trial themselves that would have increased their control, which could have reduced the performance decline.

Signs of longer-term fatigue were also shown for the TOJ threshold measurements: Thresholds increased over the whole course of the sixteen consecutive measurements despite that participants were allowed to rest between blocks. It is possible that this longer-term threshold increase is based on the same temporal-processing specific mechanisms as was suggested above for the short-term fatigue phenomenon. However, it is also possible that the longer-term effect has a different source. Note that because some of the participants did rest between the conditions, fully continuous 16 measurements of TOJ would provide a better assessment of the long-term fatigue effect.

In the pilot Experiment (see Suppl. Section A) as well as in Experiment II, the main effects of pause and feedback positively correlated, and the effects of the additional manipulations were negatively related to each other (e.g., when pause had a large effect, an additional feedback resulted in a smaller increase in performance than when the pause effect was small; see Table 1). This suggests a common source of residual capacity that can be mobilized by either manipulation.

The fact that the short-term performance deterioration cannot be explained by a lack of willingness to perform the task does not mean that it is not susceptible to motivational factors. While performance decline was present when only the participant’s actual performance was presented as feedback (Simon and Winkler 2018), the evaluation of performance compared to some “standard” eliminated the performance decline, as proximity to a meaningful “standard” increases competitiveness (Garcia et al. 2006).

While TOJ thresholds did not initially differ between the two groups of participants (i.e., similar first TOJ thresholds), the groups with different feedback valence schedule differed in their trajectory of performance change. This can be explained by assuming that the influence of negative and positive feedback on arousal differ from each other (Venables and Fairclough 2009). The effect of feedback valence was not local, as we found no effect specific to the valence of the very first feedback received by the participant. Rather, possibly the overall amount of negative feedback or the overall ratio between the two types of feedback matters, since the group receiving negative feedback after the first measurement received more negative feedback overall than the other group (and no positive feedback). This explanation is also supported by the finding that the same group difference (“negative start” group advantage) was found in the no-pause/no-feedback (NP-NF) condition in which participants did not receive any actual feedback, because the NP-NF condition appeared after one or both feedback conditions for many of the participants. Alternatively, one could hypothesize that the participant’s view of the experiment differed between the groups due to the different feedback protocols. However, this explanation is incompatible with the finding that no significant difference was found between the two groups in their motivation to perform the task.

The current objective index of fatigue (performance decline) showed no correlation with subjective fatigue. Subjective fatigue correlated with required effort, initial fatigue (Experiment III), sensitivity to criticism (Experiment II) and the attenuation of positive affectivity (Experiments I and III). This result is in line with the prediction from the literature that the two measures (objective and subjective indices of fatigue) are frequently unrelated (Ackerman and Kanfer 2009; Leavitt and DeLuca 2010; Gergelyfi et al. 2015; Hornsby et al. 2016; Takács et al. 2019), as subjective fatigue is rather an adaptive signal that reflects the cost/benefit evaluation of continuing to perform a task (Hockey 1997; Boksem and Tops 2008) rather than the actual physiological/source depletion.

Measuring the TOJ threshold multiple times allows the examination of performance deterioration within a short period of time, a putative fatigue effect. This is a promising prospect, given that an average fatigue study requires several hours without a guarantee for observing a fatigue effect (Park et al. 2001; Ackerman and Kanfer 2009; Hopstaken et al. 2015a, b). It should be noted that some studies based on the strength model of self-control (Baumeister et al. 2007) claim to have found performance degradation after a few minutes of a depleting task. However, more recent studies argue that no meaningful effect can be found in these kinds of paradigms (Carter and McCullough 2014; Hagger et al. 2016). Therefore, the TOJ task may become a useful tool of assessing fatigue. The current study also found some good candidates predictors of subjective fatigue, which can be explored in more detail in longer sessions.

## Electronic supplementary material

Below is the link to the electronic supplementary material.

Supplementary file1 (DOCX 1014 kb)
