# Supplementary material for: Short-term cognitive fatigue effect on auditory temporal order judgments
Source: Exp Brain Res. 2020 Jan 3;238(2):305–19. doi: 10.1007/s00221-019-05712-x (PMC7007914; doi:10.1007/s00221-019-05712-x)
Supplement: Supplementary file 1 — Supplementary file1 (DOCX 1014 kb) [file 221_2019_5712_MOESM1_ESM.docx]

**Electronic Supplementary Material**

To “Short-term fatigue effect on auditory temporal order judgments ”, written by Júlia Simon, Endre Takács, Gábor Orosz, Borbála Berki and István Winkler

Experimental Brain Research

Corresponding author: Júlia Simon

ORCID: 0000-0001-9251-5527

Institute of Cognitive Neuroscience and Psychology, Research Centre for Natural Sciences, Hungarian Academy of Sciences, Budapest, Hungary;

Department of Cognitive Science, Faculty of Natural Sciences, Budapest University of Technology and Economics, Budapest, Hungary;

Faculty of Humanities and Social Sciences, Pázmány Péter Catholic University, Hungary

Contact information: e-mail: simon.julia@ttk.mta.hu, simon.julia89@gmail.com

Address: 1117 Budapest, Magyar tudósok körútja 2.

Phone number: +36 1 382 6809, +36 30 9237539

**A)**

**1. Pilot Experiment**

Firstly, we tested whether the quick performance decline can be replicated. Therefore, the experiment started with four consecutive threshold measurements without feedback or mandatory pauses. Furthermore, we hypothesized that the objective fatigue effect could be eliminated or attenuated with either mandatory pauses or motivating feedback. In Simon and Winkler’s (2018) study feedback consisted of presenting the achieved threshold to the participant. In this pilot experiment, participants also received information whether the latest performance was better or worse than the average threshold of young adults. It is assumed, that this type of feedback will be more motivating than the simple feedback of the previous study (Garcia, Tor, & Gonzalez, 2006). A 2x2 design was employed for manipulating these variables. The design also allowed testing whether the two variables (rest between measures and feedback) act independently or activate the same residual resources. Therefore, we analyzed the correlations between the manipulation effects.

In order to test whether participants experience stress during the measurements, which could be detrimental to their performance, their stress level was measured with a short questionnaire. As performance-related stress might be more predictive than general stress, perfectionism was also measured (Rice, Richardson, & Tueller, 2013; translated by Orosz and Tóth-Király, personal communication). Also, to test the potential change in task commitment an Achievement Goals Questionnaire (Elliot, Murayama, and Pekrun, 2011; Urban et al., 2014) was applied three times during the experiment.

In order to test the task-specificity of the effect (whether a comparable performance decline can be detected with other auditory tasks given our participant pool), a Compressed Speech Task (CST) was also measured three times in a row. Given the implication of the role of the threshold measured by TOJ in speech processing (Tallal et al., 1998), possible links between the two tasks (TOJ and CST) were also explored.

**2. Methods**

**2.1. Participants**

The experiment included 32 healthy, native Hungarian speaking young adults (age 19-27, 17 females), 81.3% right-handed and 50% had practiced music for a longer period of time. All participants had a hearing threshold below 20 dB SPL at 1000 Hz and the difference between the ears did not exceed 10 dB. Each subject provided written informed consent to procedures approved by the United Ethical Review Committee for Research in Psychology (EPKEB) and they received a modest financial compensation for their participation through a student work organization.

The experiment took place in the laboratory of the Institute of Cognitive Neuroscience and Psychology of the Research Centre for Natural Sciences of the Hungarian Academy of Sciences..

**2.2. Stimuli and procedures**

The same TOJ paradigm and equipment was used as in the first experiment.

2.2.1. *Compressed Speech test.* We recorded 80 pseudo words of CVCCV structure with similar phonotactic properties (see Supplementary Material Section B) with a 44100 Hz sampling rate at 24 bits using an Audio Technica 2020 microphone and an EIE Pro Sound Card. The words were spoken three times by a native Hungarian male speaker. The second of the three utterances was selected, because in general, it had the most natural-like prosody. For each listener, a random half of the words was compressed by deleting without replacement 7 segments of 200 data points from the overall 40000 data points. The whole signal was segmented into 200 epochs, a random 7 of which were deleted. Thus overall 3.5% of the signal was removed. This method was inspired by Jafari et al. (2013); however, in contrast to Jafari et al. (2013) we presented two syllable words and 3.5% compression rate, which was established *based on pilot* in which we established that neither ceiling nor floor effect was reached with the compressed words. The compressed and normal words were presented in a pseudorandom order and participants had to type in the words they heard using the keyboard of a personal computer. Their answers appeared on the screen and the participants were able to edit them until they hit ENTER. All words (N = 80) were delivered three times. One test block lasted about 6.5 minutes. Because of this, two Compressed Speech tests in a row last longer than four TOJ threshold measurements. With three successive tests any time-on-task related performance decrement should be detectable.

2.2.2. *Achievement Goal Questionnaire (AGQ).* This is an adapted version of the Elliot, Murayama and Pekrun (2011) questionnaire by Urban et al. (2014). It consists of 18 items evaluated on a 7 point Likert Scale (e.g., item: ‘My goal was to perform well in the task.’; response alternatives: 1 – ‘Not true in my case’ to 7 – ‘Excessively true in my case’). The following subscale scores can be computed: Task-approach, Task-avoidance (in the sense that someone wanted to avoid failure in the task), Other-approach, Other-avoidance, Self-approach and Self-avoidance. In this study, we focused on the first four subscales to assess the consistency in the motivation to perform well in the different tasks (Task-goals) and to test the effects of our manipulations. The AGQ was measured three times: after the first TOJ, after two blocks (in the middle) of the four TOJ blocks (see the Procedure section), and at the end of the experiment.

2.2.3. *Experimental Stress Questionnaire*. The participants responded to 10 items using a 5 point Likert scale (0 – ‘Never’, 4 – ‘Very often’). Items were related to the frustration experienced during the experiment (e.g. ‘How frequently you felt nervous or stressed?’). The test was developed by Orosz and Tóth-Király (personal communication, see in Supplementary Material Section D) and the items can be found in the Supplement with non-validated translations. The Cronbach’s Alpha was .752 in our sample.

2.2.4. *Short form of the Revised Almost Perfect Scale* (Rice, Richardson & Tueller, 2013). The Hungarian version (Orosz and Tóth-Király, personal communication, see in Supplementary Material Section D) of the scale was employed. The participants marked how well each of the 23 items applied to them on a 7 point Likert scale (1 – ‘Strongly disagree’, 7 – ‘Strongly agree’). The scale has two main subscales: Standards (high performance expectations, 7 items) and Discrepancy (self-critical performance evaluations, 12 items). The Cronbach’s alpha of the Standard subscale was .796 and of the Discrepancy subscale was .905 in this study.

2.2.5. *Procedure*

After the practice period (see in the Methods of the Initial findings section), participants completed four TOJ runs without feedback or mandatory pauses (condition C0). This was followed by three runs of the Hungarian Compressed Speech task. In the second phase of the experiment, the order of the conditions (one block with 4 runs of TOJ threshold measurement, each) of the 2x2 design (N = 4) was randomized. The four conditions are: F-P (condition with feedback and mandatory pauses between the measurements), F – NP (condition with feedback but without mandatory pause), NF – P (condition without feedback but with mandatory pauses) and NF – NP (a condition without feedback or mandatory pauses). At the beginning of this part, participants were informed that performing above average would be rewarded. Based on the Simon and Winkler’s (2018) experiment, the average was 45 ms, and values lower than 45 were regarded as better than average performance. The structure of the experimental session is shown in Supplementary Figure 1.


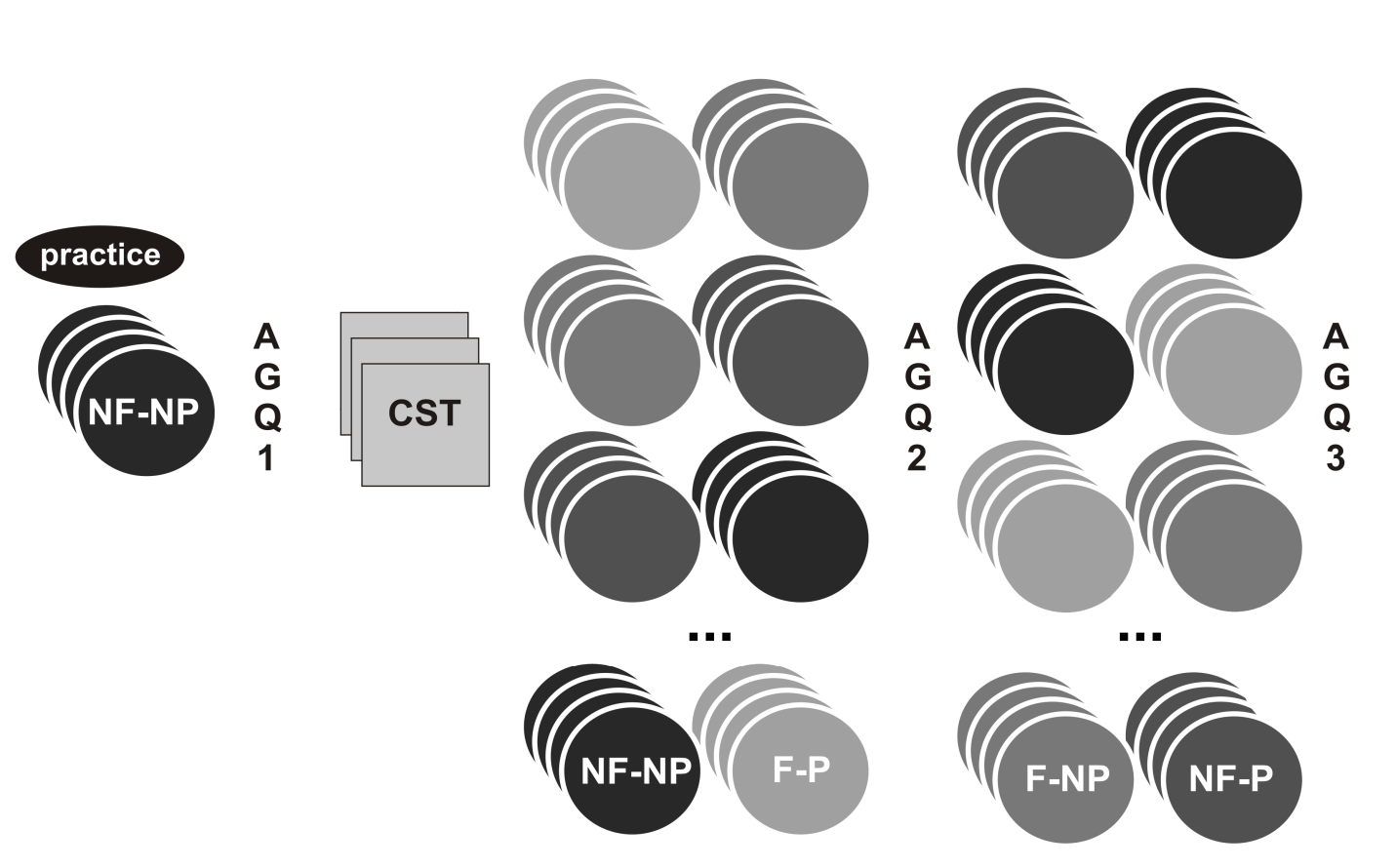


**Supplementary Fig.1**. The structure of the experimental tasks. Each stack of circles in the figure represents one block of TOJ threshold measurements (four consecutive identical measurements). The grey shade of the circle marks the condition, where F/NP refers to “feedback/no feedback”, while P/NP to “pause/no pause”. Participant performed the 4 conditions in a different order. AGQ marks filling the Achievement Goal Questionnaire, the number following the abbreviation denotes the 1^st^, 2^nd^, and 3^rd^ assessment of achievements goals. The “CST” stack of squares represents the 3 consecutive measurements of the Compressed Speech Test.

In two conditions, mandatory pauses were inserted between runs: for one minute, a picture of mountains appeared and classical music was played (see a more detailed description in the main test: Section 3.1.2.1.). During the breaks between blocks (corresponding to a condition), the subjects were required to rest and in order to facilitate the regeneration process, water, sweets and crackers were put on the table.

In two conditions, feedback was provided after each threshold measurement. Whenever there was a mandatory pause inserted, the feedback to the previous measurement was presented after this rest period. The feedback was false; its three variants were delivered in random order across the four runs.

Neutral: ‘Your latest threshold is X+e ms. The average threshold of young adult’s is 45 ms. You performed better than the average with e ms.’ (2<e<3 ms)

Positive: ‘Your latest threshold is X-e ms. The average threshold of young adult’s is 45 ms. You performed better than the average with e ms.’ (20<e<25 ms)

Negative: ‘Your latest threshold is X+e ms. The average threshold of young adult’s is 45 ms. You performed worse than the average with e ms.’ (20<e<25 ms)

After the fourth measurement there was no performance related feedback.

After the completion of all tasks, all participants received a reward in the form of a chocolate bar irrespective of their performance and the false feedbacks received.

2.2.6. *Statistical analyzes*.

In the descriptive statistics, the average TOJ threshold of the condition is based on the average of the four measures except the first one that is not influenced by the condition manipulation.

The effect of repetitions was tested with a repeated measures ANOVA having the factors RUN (the four consecutive measurements) and CONTEXT (N = 2, beginning [C0] vs. the end of the experimental session [C1/NF-NP]). Post hoc pairwise comparisons were Bonferroni-corrected.

In order to test the manipulation effects a repeated measures ANOVA was run with the factors RUN (N = 3, the first run was omitted from these analysis as it is not affected by either manipulation, not having been preceded by a pause or feedback), PAUSE (N = 2, with or without a mandatory pause) and FEEDBACK (N = 2, with or without feedback).

The two manipulation effects were correlated (Spearman’s Rank correlation) in order to test whether they mobilized the same residual capacity or not.

The feedback valence effect was tested with a repeated measures ANOVA having the factors PAUSE (N = 2; condition with and without mandatory rest periods) and VALENCE (N = 3; neutral, positive and negative) on the TOJ threshold measured from the run following the given type of feedback (either the 2^nd^, the 3^rd^ or the 4^th^ run, randomly varying across participants).

The potential change in the percentage of the correct responses in the CST task was examined with a repeated measures ANOVA having the factors COMPRESSION (compressed versus uncompressed) and RUN (N=3; first, second and third repetition of the same set).

The potential change in the Achievements Goals was tested with a repeated measures ANOVA having the factors TYPE (N = 2; Task-goals or Other-goals), QUALITY (N = 2; approach or avoidance) and RUN (N = 3; the three repetition). We expected no change in the Task-goals but an increase in the Other-goals due to the feedback manipulation.

All other statistical testing was conducted as described for the Initial findings.

**3. Results**

The data is available on the following site: <https://osf.io/2pbck/>

*TOJ thresholds.* The average thresholds in the five different conditions were the following: at C01-4 M = 45.067 ms (SD = 24.99, range 6.56-117.5), at C1/NF-NP2-4: M = 61.95 (SD = 29.99, range 14.58-119.17), at NF-P2-4: M = 52.06 (SD = 24.39, range 6.33-118.33), at F-P2-4: M = 49.19 (SD = 21.97, range 5.42-86.11) and at F-NP2-4: M = 52.71 (SD = 24.51, range 10.44-105.83). The changes over the course of the four runs are shown in Figure 2 and Figure 3. The cross-correlations between TOJ threshold measurements can be examined in the Supplementary Material Section C Table 3.


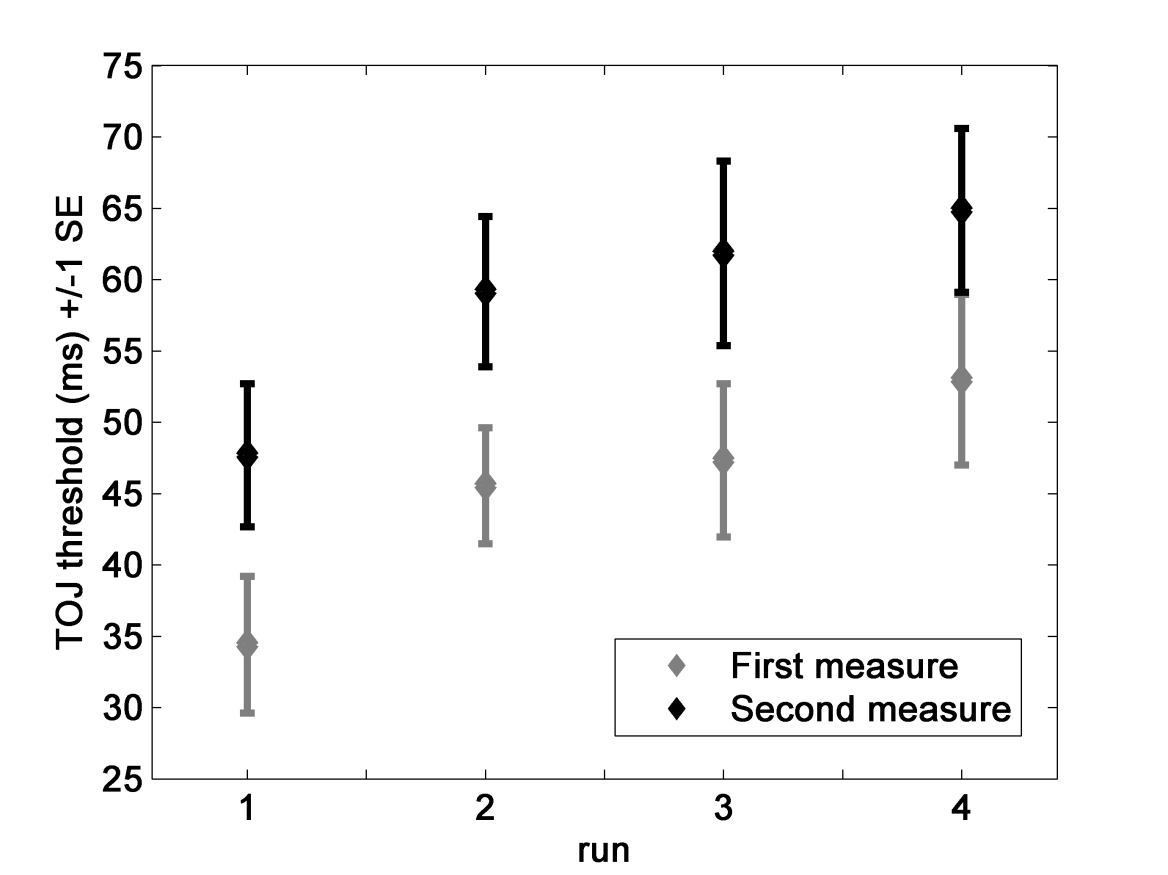


**Supplementary Fig. 2.** A gradual decline in TOJ threshold performance is shown without mandatory pause and feedback. The first measure was taken at the beginning of the experimental session, the second during the TOJ measurements in the second part of the session (see Supplementary Figure 1). The means are presented with +/-1 SE (standard error).

A two-way repeated measures ANOVA with factors of RUN (N = 4) and CONTEXT (N = 2, C0 vs. C1/NF-NP) yielded a significant main effect of RUN (F(3,93) = 11.199, MSE = 332.110, p < .001, pη^2^ = .265). All comparisons were significant, except the difference between runs 2 and 3 also between 3 and 4. The main effect of CONTEXT was significant (F(1,31) = 12.424, MSE = 914.574, p = .001, pη^2^ = .286), suggesting an overall better performance at the beginning than at the end of the experiment. There was no significant interaction between the two factors (p = .974).

A three-way repeated measures ANOVA of the effects of experimental manipulations (Figure 3) with factors of RUN (N = 3), PAUSE (N = 2, with or without a mandatory pause), and FEEDBACK ( N = 2, with or without feedback) showed a main effect of PAUSE (F(1,31) = 11.666, MSE = 370.123, p = .002, pη^2^ = 0.273) and FEEDBACK (F(1,31) = 5.173, MSE = 616.345, p = .023, pη^2^ = .156), but no significant interactions or main effect of RUN (all p > .26). Both feedback and pause decreased the threshold (improved the performance).


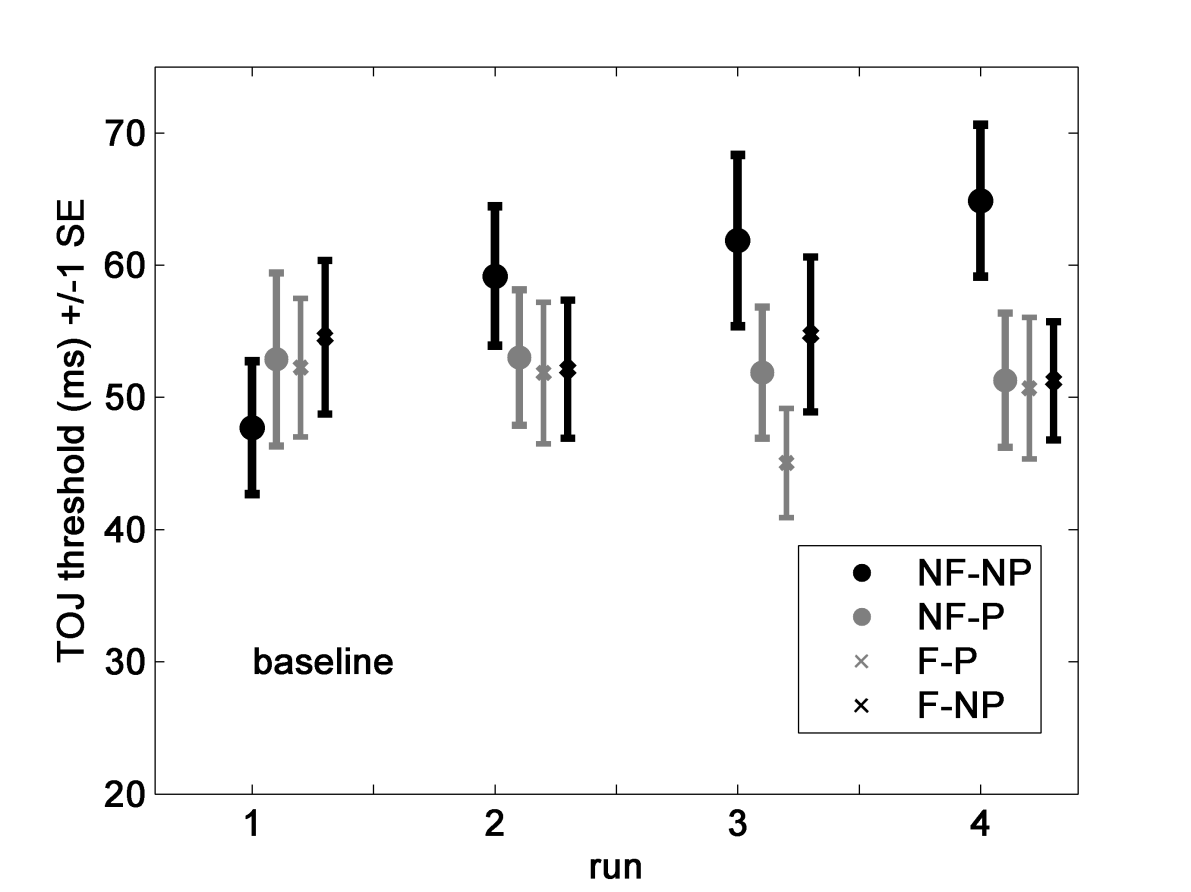


**Supplementary Fig. 3.** The mean TOJ thresholds (with +/-1 SE) in the four conditions of the 2x2 design. The first run is considered baseline as it was not preceded by either a pause or feedback. The thick black markers correspond to the NF-NP (no feedback, no mandatory pause) condition showing higher thresholds for repeated runs than any other condition.

The pause effect was 9.88 ms (NF-NP minus NF-P), the feedback effect was 9.23 ms (NF-NP minus F-NP), and the joined effect 12.75 ms (NF-NP minus F-P) with 3.52 ms additional pause effect on top of the feedback effect (F-NP minus F-P) and 2.87 ms additional feedback effect (P-NF minus P-F). The correlations between the pause and feedback effects (both direct and additional) are summarized in Table 1.

*Supplementary Table 1.* The rho correlation coefficients calculated between the various manipulation effects. The abbreviation ‘Add.’ refers additional effects, see main text.

|  | **Feedback** | **Add. Pause** | **Add. Feedback** |
| --- | --- | --- | --- |
| **Pause** | **.678** (p < .001) | -.256 (p >.05) | **-.439** (p = .012) |
| **Feedback** |  | **-.538** (p < .001) | -.176 (p > .05) |
| **Add. Pause** |  |  | **.602** (p < .001) |

The feedback valence effect was examined with a two-way repeated measures ANOVA with the factors PAUSE (N = 2) and VALENCE (N = 3; neutral, positive and negative). No main effect or interaction turned out to be significant (all p > .13).

*Compressed Speech test.* Accuracy (percentage of correct responses) as a function of runs did not change for control words (M1 = .941, M2 = .946, M3 = .945), but increased for compressed words: M1 = .503 (range .2-.78), M2 = .583 (.3-.8), M3 = .592 (.35-.85); see also Figure 4. A repeated measures ANOVA of the percentages of the correct responses was conducted with the factors COMPRESSION (compressed versus normal) and RUN (N=3). All main effects and interactions were significant: COMPRESSION (F(1,31) = 401.00, MSE = .018, p < .001, pη^2^ = .928), RUN (F(1.678,52.029) = 26.911, MSE = .002, p < .001, pη^2^ = .465), and COMPRESSION × RUN (F(2,62) = 22.275, MSE = .002, p < .001, pη^2^ = .418).


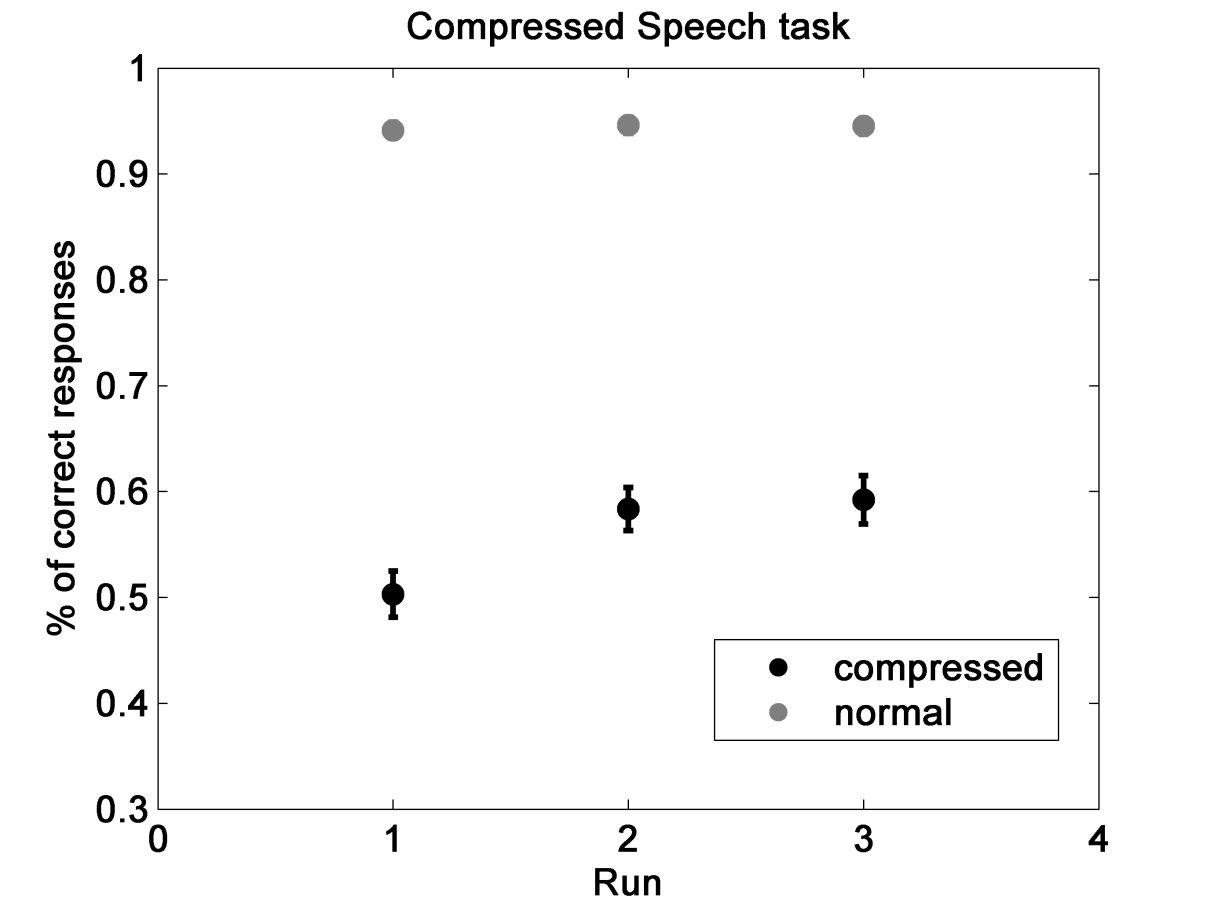


**Supplementary Fig. 4.** Accuracy in the Compressed Speech task. Black markers indicate the average (+/- 1 SE) percentage of the correct identification of ‘compressed words’ grey markers for control words.

*Correlations between the results of the Compressed Speech Test and TOJ thresholds*. The correlations are summarized in Table 2.

*Supplementary Table 2.* The rho coefficients and their corresponding p values of the correlations between the CST and TOJ task performances. C0 det. – performance deterioration in the first four measurements (4 minus 1), C1 det. – performance deterioration in the NF-NP condition from the second part of the experiment, Mean C0 – the average TOJ threshold based on the first four measurements, Comp. – accuracy in the compressed trials, Uncomp. – uncompressed trials, Comp2-1 – accuracy difference between the second and first measures.

|  | Comp. 1 | Comp. 2 | Comp. 3 | Uncomp 1 | Comp2-1 | Comp3-1 |
| --- | --- | --- | --- | --- | --- | --- |
| C0 det. | -.070  .703 | -.023  .903 | .013  .942 | -.263  .147 | .258  .154 | .233  .199 |
| C1 det. | -.**425**  .015 | -.**432**  .014 | -.**477**  .006 | -.139  .448 | -.129  .481 | -.084  .646 |
| Mean C0 | -.130  .477 | -.130  .477 | -.111  .547 | -.159  .385 | .053  .772 | .023  .899 |

*Achievement Goal Questionnaire.* The repeated measure ANOVA having the within subject factors TYPE (N = 2: Task-goals, Other-goals), RUN (N = 3) and QUALITY (N = 2; approach or avoidance) showed a main effect of TYPE (F(31) = 31.00, MSE = 8.142, p < .001, pη^2^ = .500), participants having higher Task-goals than Other-goals. There was also a main effect of RUN (F(2,45.994) = 6.10, MSE = 1.227, p = .009, ε = .742, pη^2^ = .164) that reflected an overall increase in motivation and an interaction effect of TYPE × RUN (F(2,47.681) = 4.86, MSE = .689, p = .019, ε = .769, pη^2^ = .136). There were no significant changes through the three measurements in the Task-goals (p = .153; M1 = 5.82 (3.5-7), M2 = 5.91 (3.83-7), M3 = 5.97 (3.67-7)), but a significant increase in the Other-goals subscale (p = .011; M1 = 3.86 (1-6.83), M2 = 4.47 (1-7), M3 = 4.48 (1-7)) (see also Supplementary Material Section C Figure 5). Finally, there was a main effect of QUALITY × RUN (F(62) = 12.23, MSE = 1.908, p = .001, pη^2^ = .283). From the second to the third measurement, the approach goals decreased and the avoidance goals increased. No other main or interaction effects were found.

The TOJ performance deterioration did not show significant correlation with any of the achievement goals or their change (see Supplementary Material Section C Table 4 and 5).

*Experimental Stress Questionnaire*. The mean score was 1.36 (SD = .48, minimum = .4, maximum = 2.3; the theoretical maximum was 4). The results of this questionnaire did not show significant correlation either with TOJ performance deterioration or with the manipulation effects (pause, feedback) (see Supplementary Material Section C Table 6).

*Revised Almost Perfect Scale*. There was a medium correlation between the two subscales (Standards and Discrepancy) (r_s_(30) = .499, p = .004). Neither subscale was significantly predictive for the TOJ performance (mean, condition effects, performance deterioration, see Supplementary Material Section C Table 7). The discrepancy subscale had a positive correlation with the of the Experimental Stress score (r_s_(30) = .479, p = .005).

**4. Discussion**

We replicated the previously found short-term performance deterioration in the TOJ task. This effect could be eliminated with mandatory pauses or comparative feedback. The simple threshold presentation was not effective in the initial experiment but an evaluation of the performance as worse or better than average turned out to be efficient. The correlations of the manipulation effects suggest the existence of an absolute threshold as the additional pause effect negatively correlate with the feedback effect and the additional feedback effect negatively correlate with the pause effect. Furthermore, the pause and feedback effect show a strong positive correlation that can signal the exploitation of a common residual capacity

Neither the average TOJ task performance, nor its deterioration show significant correlation with the measured experimental stress, perfectionism or achievement goals. The lack of these correlations together suggests that the observed performance deterioration is a genuine fatigue effect instead of a change in dispositions. In fact, the task motivation as measured by the AGQ Questionnaire even increased during the experiment, especially the avoidance in the Other-goals factor (i.e., participants did not want to perform worse than others), which may reflect an effective feedback manipulation.

The measured increase in the CST task also argues against a nonspecific time-on-task effect, like decreased willingness to pay attention. However, the CST task might have been more interesting to the participants than the TOJ task. Therefore, a comparison with the results of an attention task would be required in the future.

Interestingly, there was a negative correlation between the CST accuracy and performance deterioration in the manipulation free condition (a better performance in speech perception predicts less performance deterioration in the TOJ task). However, this relationship was not found in relation with the performance deterioration in the very first block of four TOJ measurements. Therefore, no clear relationship can be identified between the two tasks.

**Supplementary References**

Jafari Z, Omidvar S, Jafarloo F (2013). Effects of ageing on speed and temporal resolution of speech stimuli in older adults. *Med J Islam Repub Iran*.27 (4) :195-203

Rice, K., Richardson, C., & Tueller, S. (2013). The Short Form of the Revised Almost Perfect Scale. *Journal of Personality Assessment*, *96*(3), 368–379. doi:10.1080/00223891.2013.838172

**B)**

**Pseudo words in the CST:**

Kamla, kamdu, kangu, karbe, kemga, kemgu, kamde, kerga, kerdo, kombu, konle, konbu, kongu, kunla, kunbo, kunga, tambo, tange, terba, tarbe, tarbo, temlu, tomlu, tomga, tonbe, turge, pamle, pamlo, pagme, pagma, panbu, parbe, parbu, pemda, perda, ponle, ponlu, porbu, punbo, purge, kalma, kadmu, kagnu, kabre, kegma, kegmu, kadme, kegra, kedro, kombu, kolne, kobnu, kognu, kulna, kubno, kugna, tabmo, tagne, tebra, tabre, tabro, telmu, tolmu, togma, tobne, tugre, palme, palmo, pamge, pamga, pabnu, pabre, pabru, pedma, pedra, polne, polnu, pobru, pubno, pugre

**C)**


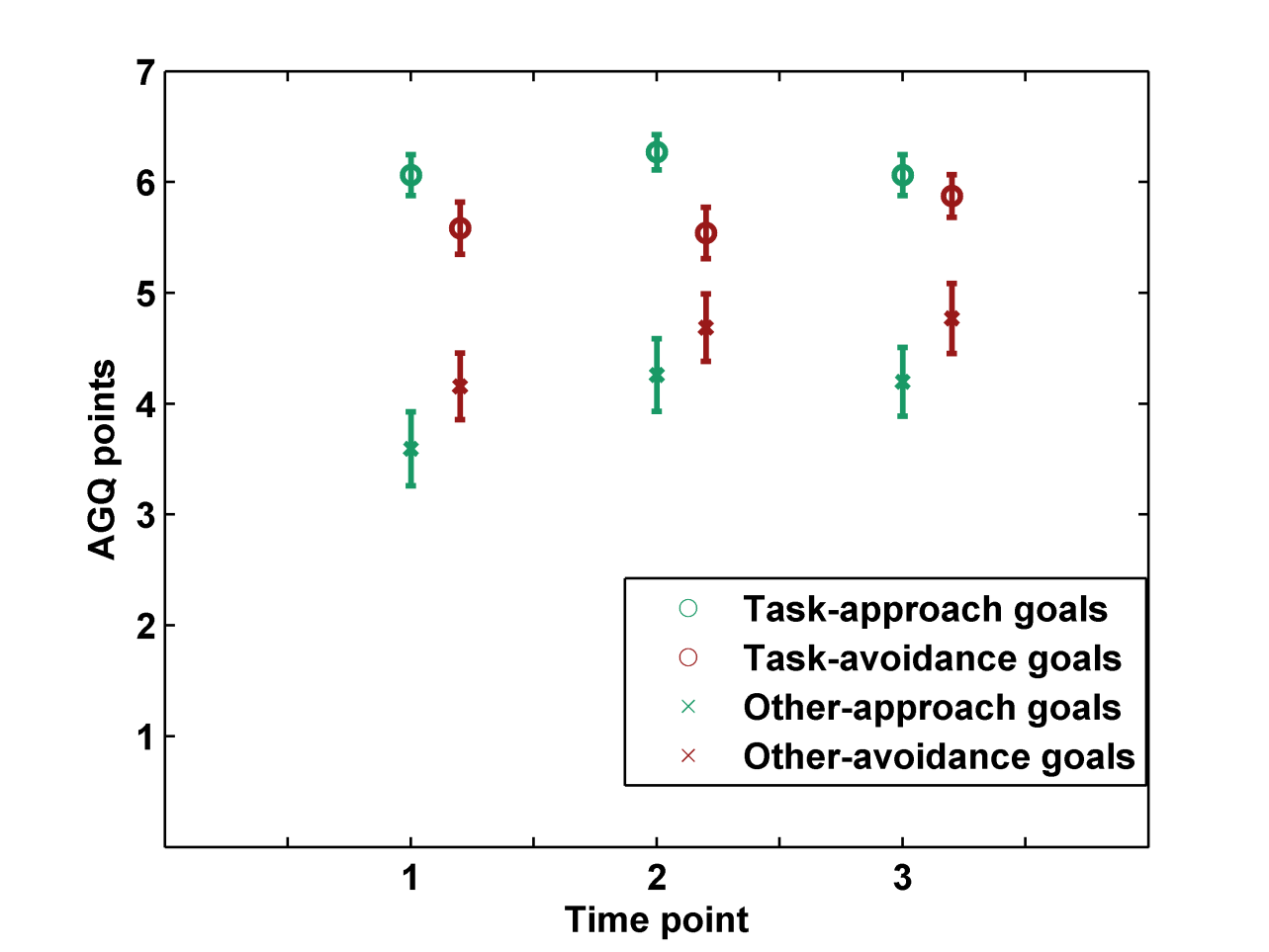


**Supplementary Fig. 5.** The results of the Hungarian 3x2 Achievement Goal Questionnaire in three time points (1 - after the first four measurements, 2 - after two blocks of the randomized four conditions and 3 - at the end of the experiment). The maximum point is 7, therefore it can be seen that the participants reported high Task-orientation.


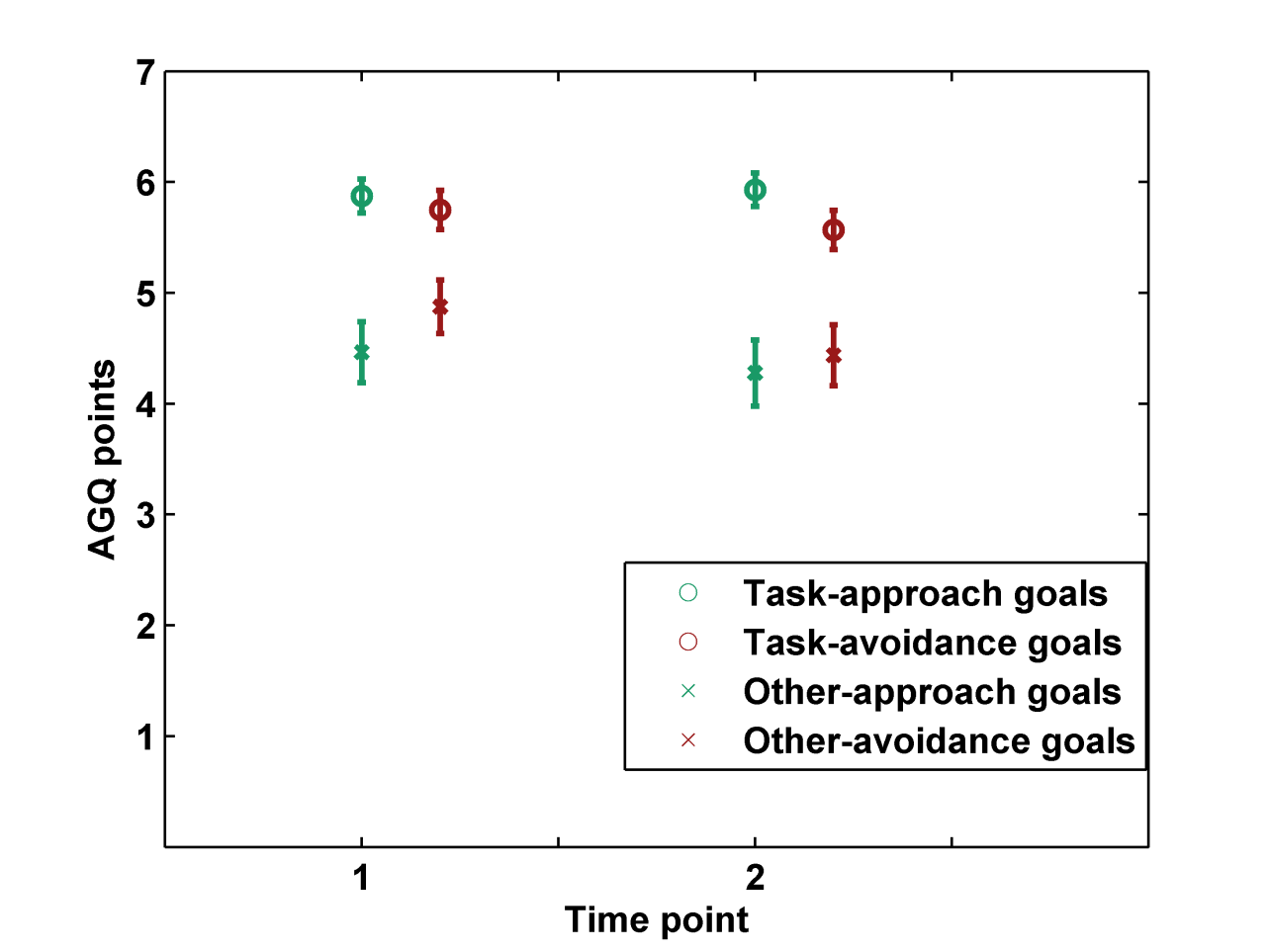


**Supplementary Fig. 6.** The results of the Hungarian 3x2 Achievement Goal Questionnaire at two time points (1 - after the TOJ measurements and 2 - after the Flanker task). The maximum point is 7, therefore it can be seen that the participants reported high Task-orientation.

Supplementary Table 3. Spearman correlation coefficients (rho values) between MTOJ measurements. The bold numbers indicate significant correlations after fdr correction with q = 0.05. C0: first block, at the beginning of the experiment; NF-NP: no feedback and no mandatory pauses; NF-P: no feedback and mandatory pauses between consecutive measurements; F-P: feedback and mandatory pauses; F-NP: feedback and no mandatory pauses.


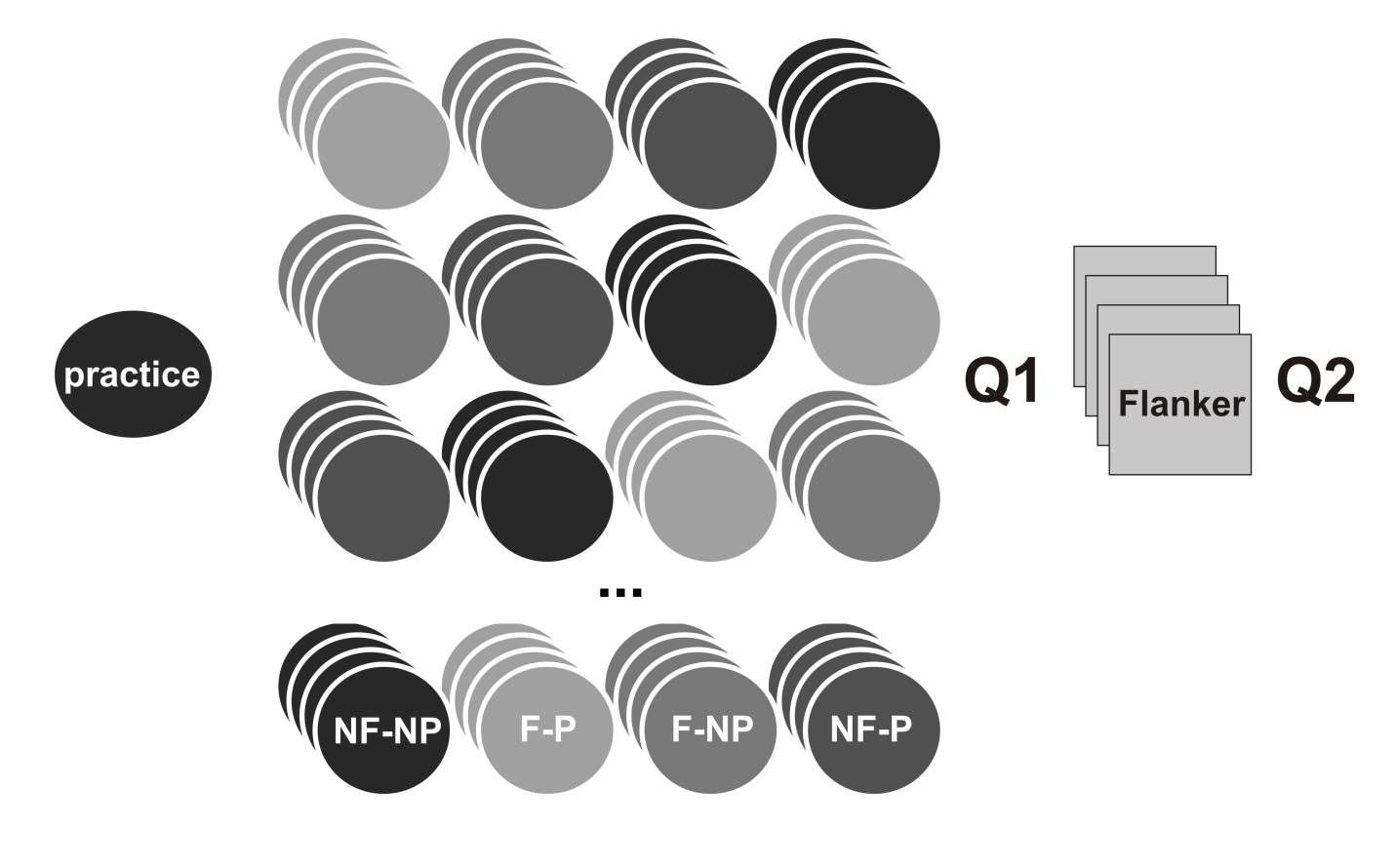


**Supplementary Fig. 7.** The structure of the experimental session. The session started with practicing the TOJ measurement task. Each stack of circles in the figure represents one block of TOJ threshold measurements (four consecutive identical measurements). The grey shade of the circle marks the condition, where F/NF refers to “feedback/no feedback”, while P/NP to “pause/no pause”. Two groups of participants’ data were collected (“positive start” vs. “negative start”) and each group consisted of 24 participants, corresponding to the 24 possible orders of the 4 different conditions (each participant of each group performed the 4 conditions in a different order). Q1 stands for the following series of questionnaires: Situational Fatigue, AGQ, Sensitivity to Criticism, Mindfulness, and Vitality/General Fatigue. Q2 marks to the second AGQ measure. The “Flanker” stack of squares represents the 4 consecutive measurements of the auditory flanker task.


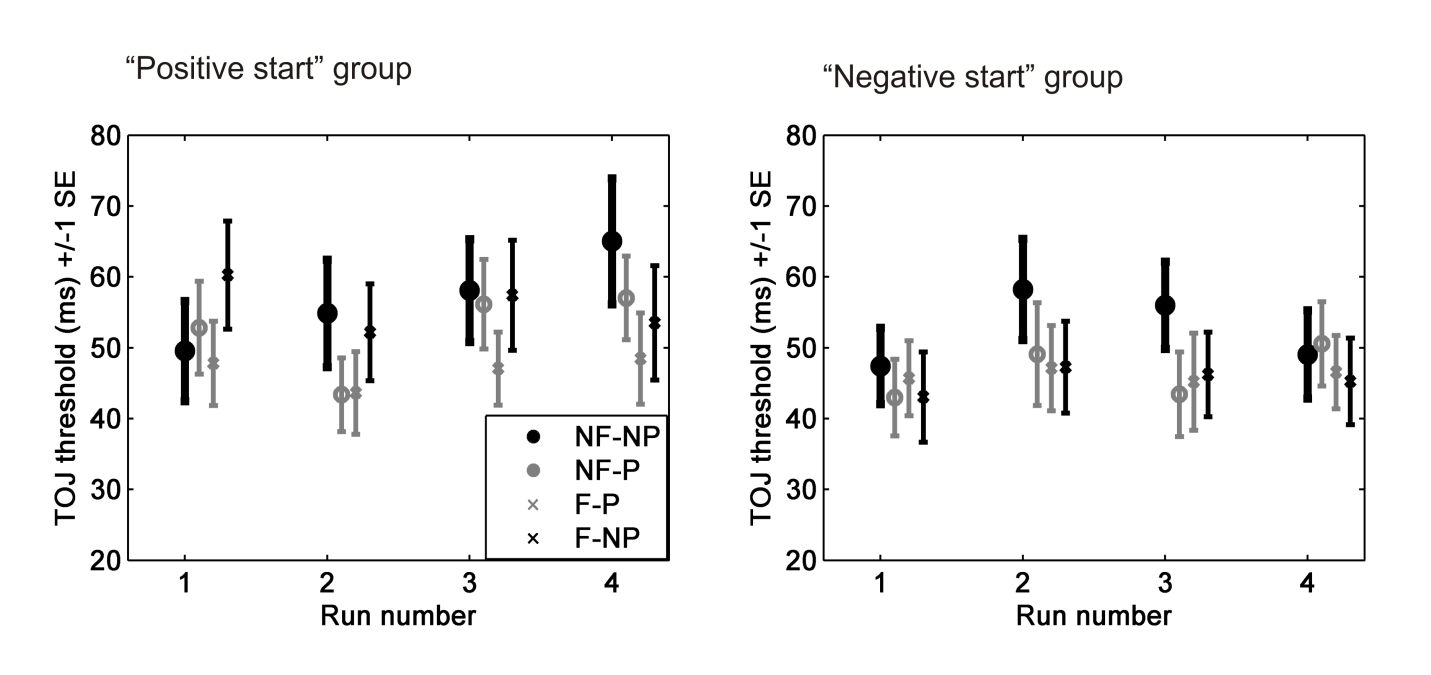


**Supplementary Fig. 8.** Group-average (N=24) TOJ thresholds (with +/-1 SE), separately for the four conditions, four runs, and two groups. The first run is considered baseline as it was not preceded by a pause or feedback. Conditions are marked by line color (grey for pause [P], black for no pause [NP]) and the center mark (“x” for feedback [F], “•” for no feedback [NF]).


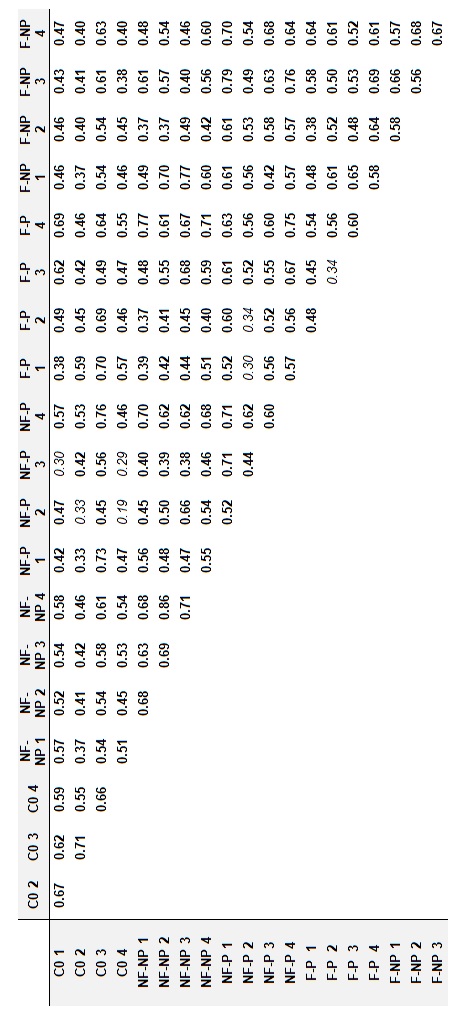


*Supplementary Table 4.* The relationship between the Achievement Goals and the objective fatigue in the TOJ task. C0 – the first block of the experiment, without feedback or mandatory pauses. NF-NP – the “no feedback, no pause” condition of the 2x2 design. 4-1 refers to the subtraction (the TOJ threshold of the last run minus the TOJ threshold of the first run). Task represents the Task Goals, Other represents the Other goals. The number represents the serial position of the measurements.

|  | **Task-1** | **Task-2** | **Task-3** | **Other-1** | **Other-2** | **Other-3** |
| --- | --- | --- | --- | --- | --- | --- |
| **C0 4-1** | -.043  p = .813 | -.127  p = .489 | -.332  p = .064 | .018  p = .921 | -.018  p = .924 | .036  p = .846 |
| **NF-NP 4-1** | -.109  p = .553 | .091  p = .622 | .080  p = .665 | -.073  p = .693 | -.116  p = .526 | .034  p = .855 |

The performance deterioration in TOJ is not related to the strength of the achievement goals.

*Supplementary Table 5.*  The manipulation effects in TOJ are not related to the change in Task-goals or Other-goals. The Spearman’s rho correlation coefficients are presented with the corresponding p value. C0 – the first block of the experiment, without feedback or mandatory pauses. NF-NP – the “no feedback, no pause” condition of the 2x2 design. 4-1 refers to the subtraction (the TOJ threshold of the last run minus the TOJ threshold of the first run).

|  | **C0 4-1** | **NF-NP 4-1** | **Pause effect** | **Pause add.** | **Feedback effect** | **Feedback add.** |
| --- | --- | --- | --- | --- | --- | --- |
| **Other 3-1** | .129  p = .483 | .287  p = .111 | .045  p = .807 | .167  p = .362 | -.107  p = .560 | -.068  p = .711 |
| **Other 3-2** | -.165  p = .367 | -.016  p = .931 | -.193  p = .289 | .032  p = .862 | -.192  p = .292 | -.342  p = .055 |
| **Task 3-1** | .116  p = .529 | .369  p = .037 | -.210  p = 250 | .073  p = .691 | -.006  p = .972 | -.083  p = .652 |
| **Task 3-2** | -.258  p = 154 | .200  p = .271 | -.191  p = .294 | .264  p = .145 | .080  p = .663 | -.370  p = .037 |

Nothing can be considered significant after correction (alpha = .002 after Bonferroni correction).

*Supplementary Table 6.* The experimental stress and the manipulation effects in the TOJ task. C0 – the first block of the experiment, without feedback or mandatory pauses. NF-NP – the “no feedback, no pause” condition of the 2x2 design. 4-1 refers to the subtraction (the TOJ threshold of the last run minus the TOJ threshold of the first run).

|  | **C0 4-1** | **NF-NP 4-1** | **Pause effect** | **Pause add.** | **Feedback effect** | **Feedback add.** |
| --- | --- | --- | --- | --- | --- | --- |
| **Stress** | -.124  p = .499 | .220  p = .226 | -.038  p = .837 | .048  p = .793 | .037  p = .840 | -.043  p = .814 |

The experimental stress did not show a significant relationship with TOJ performance deterioration or with the manipulation effects in the pilot experiment.

*Supplementary Table 7.*  The relationship of the two perfectionism subscale with the TOJ performance. C0 – the first block of the experiment, without feedback or mandatory pauses. NF-NP – the “no feedback, no pause” condition of the 2x2 design. 4-1 refers to the subtraction (the TOJ threshold of the last run minus the TOJ threshold of the first run).

|  | **Mean 16** | **C0 4-1** | **NF-NP 4-1** | **pause** | **Pause add.** | **Feedback** | **Feedback add.** |
| --- | --- | --- | --- | --- | --- | --- | --- |
| **Standard** | .145 | -.073 | .032 | -.024 | -.097 | .203 | .099 |
| **Discrepancy** | .267 | -.043 | .293 | -.013 | .014 | .201 | .050 |

There is no significant correlation even without correction between perfectionism and TOJ performance.

Supplementary Table 8. Mean (N=48) incongruent-minus-congruent differences in the Flanker task with the standard deviation in parenthesis.

|  | Run 1 | Run 2 | Run 3 | Run 4 |
| --- | --- | --- | --- | --- |
| **RT (ms)** | 85 (89) | 95 (87) | 100 (86) | 90 (97) |
| **Error (%)** | 5.0 (5.9) | 4.5 (4.6) | 6.4 (6.6) | 5.4 (5.8) |
| **Miss (%)** | 1.8 (4.3) | 1.3 (2.9) | 1.4 (3.7) | 2.1 (5.4) |

The mixed ANOVA of the miss rates showed a significant CONGRUENCY (F(1,46) = 13.8, MSE = .002, p = .001, pη^2^ = .231) but no significant RUN1-4 effect (F(3,138) = 2.518, MSE = .001, p = .061, pη^2^ = .052) or interaction.

Supplementary Table 9 and 10. Comparison between Spearman’s and Pearson’s correlation results for all correlations tested

Initial findings

|  | Correlation between variables | Pearson | Spearman |
| --- | --- | --- | --- |
| 1 | Change in subjective fatigue and change in positive affectivity | r = -.467 (p = .005) | r = -.404 (p = .016) |
| 2 | Change in subjective fatigue and change in negative affectivity | r = .261 (p = .261) | r = .192 (p = .236) |
| 3 | Change in subjective fatigue and change in objective performance (threshold4-threshold1) | r = -.039 (p = .810) | r = -.066 ( p = .688) |
| 4 | Change in objective performance and subjective fatigue at the beginning of the session. | r = .169 (p = .292) | r = .128 (p = .426) |
| 5 | Change in objective performance and subjective fatigue at the end of the session. | r = .000 (p = 1) | r = -.053 (p = .746) |

Main experiment

|  | Correlation between variables | Pearson | Spearman |
| --- | --- | --- | --- |
| 1 | Pause effect and Feedback effect | r = .277 (p = .057) | r = .357 (p = .013) |
| 2 | Pause effect and additional pause effect | r = .532 (p < .001) | r = .434 (p = .002) |
| 3 | Pause effect and additional feedback effect | r = **-.433** (p = .002) | r = **-.374** (p = .009) |
| 4 | Feedback effect and additional pause effect | r = **-.191** (p = .194) | r = **-.218** (p = .137) |
| 5 | Feedback effect and additional feedback effect | r = .205 (p = .163) | r = .156 (p = .290) |
| 6 | Additional pause and additional feedback effect | r = .294 (p = .042) | r = .309 (p = .033) |
| 7 | Subjective situational fatigue and sensitivity to criticism | r = .460 (p = .001) | r = .450 (p = .001) |
| 8 | Subjective situational fatigue and objective performance deterioration (threshold4-threshold1, NF-NP condition) | r = -.229 ( p = .117) | r = -.211 (p = .150) |

**D)**

**Additional Methods**

*Additional Questionnaires in the Main Experiment*

*Mindfulness measurement*. The Hungarian version (Orosz et al., in preparation) of the ‘Cognitive and Affective Mindfulness Scale-Revised (CAMS-R)’ was used to measure the participants’ mindfulness (Feldman et al., 2007). Ten statements should have been scored on a 4-point Likert scale (1 – ‘Rarely/Not at all’, 4 – ‘Almost always’). An example statement is: “I am able to focus on the present moment”. The 10-item version had a Cronbach’s Alpha of .802.

Feldman G, Hayes A, Kumar S, Greeson J, & Laurenceau JP (2007) Mindfulness and emotion regulation: The development and initial validation of the Cognitive and Affective Mindfulness Scale-Revised (CAMS-R). *Journal of Psychopathology and Behavioral Assessment*, *29*(3), 177-190.

*Subjective Vitality and General Subjective Fatigue*. The 7 items of Subjective Vitality Questionnaire (Ryan & Frederick, 1997) was used intermixed with the items of Subjective Fatigue Questionnaire (see the questions in D 3.5.). The latter differed from the Situational Subjective Fatigue Questionnaire only in the instruction. The subjects were told to consider their answers in regard with their experiences from the last month. The Subjective Vitality Questionnaire had a Cronbach’s Alpha of .903 (that might suggest a little redundancy in the questionnaire). The Cronbach’s Alpha of the General Subjective Fatigue Questionnaire was .675 what is below optimal and it could not been meaningfully improved by the exclusion of any of the items.

**Additional Results**

*Mindfulness*. The mindfulness scores did not show significant correlations with any of the TOJ measures of interest (mean, performance deterioration, first measure), but negatively correlated with the situational subjective fatigue (r_s_(46) = -.388, p = .019), general subjective fatigue (rs(46) = -.392, p = .006) and with the sensitivity to criticism (r_s_(46) = -.642, p < .001). However, it showed a positive correlation with general vitality (r_s_(46) = .465, p = .001).

*Fatigue and Vitality*. Furthermore, the reported general fatigue showed a negative (r_s_(46) = -.29, p = .044) and general vitality showed a positive (r_s_(46) = .344, p = .017) correlation with the objective performance deterioration. Meaning, participants considering themselves more energetic performed worse at the fourth measure compared to the first. These variables (vitality and general fatigue) were not predictive to the mean thresholds, although there was a modest positive correlation between the minimum threshold and general vitality (r_s_(46) = .343, p = .017).

**E)**

**Unpublished Questionnaires**

*1. Experimental Stress Questionnaire*

Hungarian version with non-validated translations

Instructions:

Az alábbi kérdések azokra az érzésekre és gondolatokra vonatkoznak, amelyek a feladat során tapasztalhattál. Kérjük, hogy minden egyes kérdésnél írd be, hogy a feladatok elvégzése során milyen gyakran volt jellemzõ rád az adott érzés vagy gondolat!

Néhány kérdés ugyan hasonlónak tûnik, de valójában különbözõek, ezért kérjük, hogy valamennyit külön kérdésként kezeld!

Tehát ne próbáld megszámolni, hogy hányszor érezted magad egy adott módon, hanem írd be azt a választ, ami a leginkább jellemzõnek tûnik!

Nincsenek jó vagy rossz válaszok, ezért kérlek, legyél nyitott és õszinte! (Tovább - SPACE)'

(The following questions refer to your experiences during the task. Please, respond to each question indicating how applicable was the given feeling or thought to you during task performance. Some questions seem similar but they are actually different, please try to treat them as separate questions.

Do not try to count how many times you felt as described, but chose the option most applicable.

There are no good or bad answers, therefore please be honest! Next - SPACE)

Responses:

0 – Soha, 1 - Szinte soha, 2 – Elõfordult, 3 - Elég gyakran, 4 - Nagyon gyakran

(0 – Never, 1 – Almost never, 2 – Sometimes, 3 – Often, 5 – Very often)

Items:

1. A feladat során milyen gyakran voltál feszült valamilyen váratlan esemény miatt? (How frequently were you tense because of a sudden event?)
2. A feladat során milyen gyakran érezted úgy, hogy képtelen vagy kézben tartani azokat a dolgokat, amelyek kellenek a feladat elvégzéséhez? (How frequently did you feel like you are not able to control the things necessary to resolve the tasks?)
3. A feladat során milyen gyakran érezted magad idegesnek és „stresszesnek”? (How frequently you felt nervous or stressed?)
4. A feladat során milyen gyakran bíztál magadban, hogy képes vagy megoldani az (esetlegesen) felmerülõ problémákat? (How frequently did you trust in yourself that you are able to handle the incidental problems during the tasks?)
5. A feladat során milyen gyakran érezted úgy, hogy a dolgok a kedved szerint alakulnak? (How frequently did you feel like everything progress as you like?)
6. A feladat során milyen gyakran érezted úgy, hogy nem tudsz eleget tenni a követelményeknek? (How frequently did you feel like you cannot fulfill the requirements?)
7. A feladat során milyen gyakran tudtad kezelni az (esetleges) bosszúságokat? (How frequently could you handle the incidental annoyances during the task?)
8. A feladat során milyen gyakran érezted, hogy a helyzet magaslatán állsz? (How frequently you felt like you have the control?)
9. A feladat során milyen gyakran érezted, hogy egyéb (a feladattal nem kapcsolatos) elvégzendõ feladatokon gondolkodsz? (How frequently you felt like you are thinking of tasks unrelated to the current task?)
10. A feladat során milyen gyakran érezted, hogy olyan nehézséggel találkoztál, amelyen nem tudsz úrrá lenni? (How frequently you felt you encountered a difficulty you could not handle?)

Items 4, 5, 7 and 8 should be mirrored during calculation (the given value should be subtracted from the maximum).

*2. Short Form of the Revised Almost Perfect Scale*

The English version is available (Rice, Richardson & Tueller, 2013), therefore only the Hungarian version is presented.

Instructions:

A következõ állítások az emberek saját magukkal, saját teljesítményükkel és másokkal kapcsolatos viszonyulásukat mérik.

Nincsenek jó vagy rossz válaszok. Kérem, értékeljen minden állítást! Támaszkodjon az elsõ benyomására és ne töltsön túl sok idõt az egyes állításokkal!

A válaszadáshoz használja a kijelentések alatti skálát, amely az egyes állításokkal kapcsolatos egyetértése fokára vonatkozik! Válaszként nyomja le a megfelelõ számú billentyût!(Tovább - SPACE)

(The following statements measure people’s attitude towards themselves, their performance and towards others.

There are no good or bad answers. Please, evaluate all the statements! Lean on your first impression and do not spend too much time with a single statement. Respond with the corresponding button! Next - SPACE)

Responses:

1 - Egyáltalán nem értek egyet, 2 - Nem értek egyet, 3 - Inkább nem értek egyet, 4 – Semleges, 5 - Inkább egyetértek, 6 – Egyetértek, 7 - Teljesen egyetértek

Items:

1. Magasra teszem a mércét az iskolai vagy munkahelyi teljesítményemmel kapcsolatban.
2. Rendszeretõ ember vagyok.
3. Gyakran frusztráltnak érzem magam, mert nem tudom elérni a céljaimat.
4. A rendezettség fontos számomra.
5. Ha nem vársz el sokat magadtól, soha nem leszel sikeres.
6. A legjobb teljesítményem sem látom soha elég jónak.
7. Úgy gondolom, hogy helyükre kell tenni a dolgokat.
8. Magas elvárásaim vannak magammal szemben.
9. Ritkán érem el azt a magas szintet, amit magamnak állítok fel.
10. Szeretek mindig szervezett és fegyelmezett lenni.
11. Az sem tûnik soha elégnek, amikor a legtöbbet hozom ki magamból.
12. Nagyon magas mércét állítok fel önmagam számára.
13. Soha nem vagyok elégedett azzal, amit elértem.
14. A legjobbat várom el magamtól.
15. Gyakan aggódom amiatt, hogy nem tudok megfelelni a saját elvárásaimnak.
16. A teljesítményem ritkán felel meg az önmagam által felállított mércének.
17. Még akkor sem vagyok elégedett, ha tudom, hogy a legtöbbet hoztam ki magamból.
18. Megpróbálom a legtöbbet kihozni magamból mindenben, amit csinálok.
19. Ritkán tudom elérni az önmagam által felállított magas teljesítményszintet.
20. Szinte soha nem vagyok elégedett a teljesítményemmel.
21. Szinte soha nem érzem azt, hogy amit csináltam, az elég jó.
22. Erõs szükségét érzem annak, hogy kiválóságra törekedjek.
23. Gyakran érzek csalódottságot egy feladat befejezése után, mert tudom, hogy jobban is megcsinálhattam volna.
24. *Situational Subjective Fatigue Questionnaire*

Instructions:

Mennyire voltak igazak rád a következõ állítások a feladatok végzése során? (Tovább - SPACE) (How applicable were to you the following statements?)

Responses:

1 - Egyáltalán nem volt igaz, 2 - Nem volt igaz, 3 - Inkább nem volt igaz, 4 - Igaz is volt, meg nem is, 5 - Inkább igaz volt, 6 - Igaz volt, 7 - Teljes mértékben igaz volt

(1 – Not at all true, 2 – Not true, 3 – Rather no, 4 – In between, 5 – Rather true, 6 – True, 7 – Totally true)

Items (the non-validated translations are presented in the main text):

1. Úgy éreztem, hogy ’agyilag’ kifáradtam a feladatok végzése alatt.
2. Közömbös volt számomra, hogy hogyan teljesítek.
3. 'Gyakran éreztem magam kimerültnek a feladatok végzése alatt.
4. Úgy éreztem, hogy egy idõ után\n\n már nem igazán voltam hatékony a feladatvégzésben.
5. Volt, hogy úgy éreztem, feladnám a próbálkozást.
6. Egyre nehezebb volt a feladatra koncentrálni.
7. *Sensitivity to criticism*

Instructions:

Most gondolj vissza azokra az eseményekre, amikor számodra fontos személyektõl számodra fontos dolgokban kritikát kaptál! Válaszolj a lehetõ legõszintébben az alábbi kérdésekre! Kérünk nyomj gombot annak megfelelõen, hogy milyen gyakran éled meg ilyen módon a kritikát!

A következõkben próbálj meg a lehetõ legõszintébb lenni magaddal szemben!(Tovább - SPACE)

(Now, think back to situations when you received critique in a topic relevant to you, from people important to you. Please answer most honestly to the questions! Please respond with the corresponding buttons: how frequently you experienced the critique in the presented way.)

Responses:

1 - Szinte soha (0-10%), 2 - Néha (11-35%), 3 - Valamivel kevesebbszer, mint az esetek felében, 4 - Valamivel többször, mint az esetek felében (51-65%), 5 – Legtöbbször (66-90%), 6 - Szinte mindig (91-100%)

(1 – Almost never, 2 – Sometimes, 3 – Bit less than half of the cases, 4 – Bit more than half of the cases, 5 – Often, 6 – Almost always)

Items (without translation as the reference article is under preparation):

1. 'Amikor egy számomra fontos dologban kemény kritikát kapok, akkor annyira felhúzom magam, hogy már el se jut hozzám, amit mondanak.
2. Amikor egy számomra fontos dologban kemény kritikát kapok, akkor megtalálom az egyensúlyt, ahol nem emésztem magam a kritikán, de nem is tolom el annak az élményét magamtól.
3. Amikor egy számomra fontos dologban kemény kritikát kapok, nem vagyok elnézõ magammal szemben, mivel folyamatosan azon kattogok, amiket mondtak.
4. Amikor egy számomra fontos dologban kemény kritikát kapok, akkor annyira felmérgesít az, aki a kritikát mondja, hogy már nem is érdekel, hogy mit mond.
5. Amikor egy számomra fontos dologban kemény kritikát kapok, akkor minden tõlem telhetõt megteszek, hogy megszabaduljak az ezzel kapcsolatos emlékektõl.
6. Amikor egy számomra fontos dologban kemény kritikát kapok, elítélem magam és az alkalmatlanságomon rágódom.
7. Amikor egy számomra fontos dologban kemény kritikát kapok, akkor annyira feldühít az, akitõl a kritika jön, hogy legszivesebben belefojtanám a szót.
8. Amikor egy számomra fontos dologban kemény kritikát kapok, akkor kívülrõl látom a történteket és különbözõ oldalakról tudom vizsgálni azt, anélkül, hogy rágódom az érzéseimen és gondolataimon.
9. Amikor egy számomra fontos dologban kemény kritikát kapok, akkor mindent elkövetek annak érdekében, hogy teljesen megszabaduljak a kritika fájdalmától.
10. Amikor egy számomra fontos dologban kemény kritikát kapok, akkor a kritika feldolgozását kézben tartom, de közben nem vagyok kemény magammal.
11. Amikor egy számomra fontos dologban kemény kritikát kapok,akkor minden energiám abba fektetem, hogy úgy ahogy van, kitöröljem a kritika szavait az emlékezetembõl.
12. Amikor egy számomra fontos dologban kemény kritikát kapok, bíráló vagyok magammal szemben és azon kattogok, hogy mi bennem a rossz.
13. Amikor egy számomra fontos dologban kemény kritikát kapok, ami nagyon igazságtalan is, akkor is az lebeg a szemem elõtt, hogy mit tanulhatok belõle.
14. Amikor egy számomra fontos dologban kemény kritikát kapok, amit ráadásul elképesztõ durván közölnek, akkor is azon gondolkodom, hogy mit tanulhatok belõle.
15. Amikor egy számomra fontos dologban kemény kritikát kapok, ami ezen felül olyan emberek elõtt történik,akik elõtt nagyon fontos, hogy jól szerepeljek,akkor is azt elemzem, hogy mit tudok ebbõl tanulni.
16. *Subjective Vitality and General Subjective Fatigue*

Instructions:

Mennyire voltak igazak rád a következõ állítások az utóbbi 1 HÓNAPBAN? (Tovább - SPACE)

(How much were true to you the following statements in the last 1 MONTH? (Next - SPACE))

Responses:

1 - Egyáltalán nem volt igaz, 2 - Nem volt igaz, 3 - Inkább nem volt igaz, 4 - Igaz is volt, meg nem is, 5 - Inkább igaz volt, 6 - Igaz volt, 7 - Teljes mértékben igaz volt

(1 – Not at all, 2 – Wasn’t true, 3 – Rather not true, 4 – Somewhat true, somewhat not true, 5 – Rather true, 6 – It was true, 7 – Totally true)

Items:

1. Élettel telinek és elevennek érztem magam. (I felt vivid and full of life.)
2. Gyakran éreztem magam ’agyilag’ fáradtnak. (I often felt mentally tired.)
3. Nem igazán érztem magam energikusnak. (I not really felt energetic.)
4. Néha kirobbanóan energikusnak érztem magam. (Sometimes I felt unbelievably energetic.)
5. Gyakran voltam kimerült. (I frequently was exhausted.)
6. Tele voltam energiával és életerõvel. (I was full with energy and vitality.)
7. Bizakodóan néztem minden új nap elébe. (I started each of my days hopefully.)
8. Az utóbbi idõben nem igazán voltam hatékony a feladataim elvégzésében. (Lately, I wasn’t really effective in the execution of my tasks.)
9. Majdnem mindig ébernek és élénknek érztem magam. (I almost always feel vigilant and vigorous.)

10. Közömbösebb voltam, mint korábban. (I was more indifferent than earlier.)

11. Néha azt éreztem, hogy fel kellene adnom a próbálkozást egy adott cél esetében. (Sometimes, I felt like I want to give up trying regarding a given goal.)

12. Energikusnak éreztem magam. (I felt energetic.)

13. Nehéz volt számomra egy dologra koncentrálni. (It was hard to me to concentrate on one thing.)

**E,**

Details of statistical analyzes – Main experiment

The TOJ threshold change in the ”no feedback, no pause” (NF-NP) condition as a function of the valence of the first feedback was tested by a mixed model ANOVA with the within subject factor RUN1-4 (N = 4; runs 1–4) and the between subject factor GROUP (N = 2). These tests were repeated with the mean error difference change (from the 1^st^ to the 4th) and the mean RT difference between the congruent and incongruent trials (as a proxy of the executive component of attentional control) in the flanker task as covariates to assess the influence of fatigue in executive functions on the short-term TOJ threshold change. (Throughout the statistical testing, post-hoc analyses for interactions involving the GROUP factor were conducted by separate repeated measures ANOVAs for the two groups.) The possible pretest TOJ threshold differences between the two groups were tested by an independent sample *t* test between the very first TOJ measurements, which preceded any experimental manipulation.

In the statistical analyses testing the effects of the feedback and pause manipulations, only TOJ thresholds from runs 2–4 were included, because the first run was not affected by either of these manipulations. A mixed model ANOVA of the TOJ thresholds tested the effects of the experimental manipulations with the within subject factors RUN2-4 (N = 3; runs 2, 3, and 4), PAUSE (N = 2, with vs. without a mandatory pause), and FEEDBACK (N = 2, with vs. without feedback) and the GROUP between subject variable (N = 2, positive start vs. negative start). Correlations between the effects of the two manipulations were tested with Spearman’s rank correlation.

Long-term fatigue effects on the TOJ threshold were tested by a mixed model ANOVA with the within subject factor TIME (N = 16; runs 1–16) and the between subject factor GROUP (N = 2).

Effects of the valence of the first feedback on the TOJ threshold were tested by a mixed model ANOVA with the within-subject factor ORDER (N = 2; first run vs. second run including feedback) and the between-subject factor GROUP (N = 2; “positive start” vs. “negative start”). The ORDER factor differentiated between the immediate effect of the valence of the feedback and the effect of feedback later in the sequence (no run with feedback before vs. one run with feedback before). Runs with and without “pause” were pooled together for this analysis, because they appeared in equal number as the first vs. the second TOJ measurement with feedback.

A mixed model ANOVA with the within subject factors TYPE (N = 2; Task-goals or Other-goals), QUALITY (N = 2; approach or avoidance), and REPORT-TIME (N = 2; post-TOJ vs. post-flanker), and the between subjects factor GROUP (N = 2) was performed on the AGQ measures in order to test a possible change in goals during the experimental session as a function of the feedback valence.

To test the possible fatigue effect in the flanker-task, a mixed model ANOVA was conducted, separately on the median reaction times (RT), miss rates (the number of misses divided by the number of trials – Supplementary Material after Supplementary Table 8), and error rates (the number of incorrect responses divided by the number of trials) with the within subject factors RUN1-4 (N = 4; runs 1–4) and CONGRUENCY (N = 2; congruent vs. incongruent trials) and the between subject factor GROUP (N = 2). The GROUP factor was included because the flanker task always followed the TOJ tasks and thus the experimental variables could have influenced the participants’ performance. Because the ISI in the flanker task was 100 ms and there were some participants, who had a maximum TOJ value higher than 100 ms, these participants could have influenced the statistical results obtained for the flanker performance even in the absence of executive function fatigue. Therefore, we rerun the test with the introduction of the mean of all 16 TOJ thresholds as a covariate in this analysis.

Correlations were assessed between TOJ threshold performance change between the 4^th^ and 1^st^ run in the NF-NP condition and questionnaire scores (situational subjective fatigue, sensitivity to criticism, etc., see Sections 3.1.2.2.-4. and Supplementary Material Sections D) as well as performance change in the flanker task (RT difference between congruent and incongruent trials) between the 4^th^ and 1^st^ run.

3.1.5. *Power analysis*. For the calculations, the G-Power 3.0.10 software (Faul et al, 2007; Faul et al, 2009) was used. A sample size of 46 is required to detect an effect of the size obtained for the FEEDBACK main effect in the pilot experiment (see Supplementary Material Section A; alpha = 0.05, power 95%, repetitions = 3, number of groups = 2). Due to design features (two groups, counterbalanced order) 48 was the nearest number meeting all criteria.

Faul F, Erdfelder E, Lang AG, & Buchner A (2007) G*Power 3: A flexible statistical power analysis program for the social, behavioral, and biomedical sciences. *Behavior Research Methods*, *39*, 175-191

Faul F, Erdfelder E, Buchner A, & Lang AG (2009) Statistical power analyses using G*Power 3.1: Tests for correlation and regression analyses. *Behavior Research Methods*, *41*, 1149-1160.

**F**,

*Sensitivity to criticism*. This scale contains a total of three factors designed to assess one’s criticism-related over engagement, disengagement, and constructive engagement. *Over engagement* refers to the experiences of getting preoccupied with and overtaken by negative emotions, rumination and worrying in the case of a negative experience (Hayes & Feldman, 2004). Its opposite might be *disengagement*, referring to the intention of suppressing or erasing the memories related to the negative experience of criticism (Hayes & Feldman, 2004). Finally, *constructive engagement* involves keeping a healthy distance from the negative experience and neither being overwhelmed by, nor being avoidant of it. Participants are asked to rate on a 6 point Likert scale the (1 – ‘Almost never (in 0-10% of cases)’ to 6 – ‘Almost always (in 91-100% of cases)’) statements, such as ‘*When I receive a self-relevant critique* *I’m not forgiving myself, because I constantly think about what was said to me*’ (see also in the Supplementary Material Section E/4). Each of the three forms of engagement is assessed with three items within the questionnaire.

Hayes, A. M., & Feldman, G. (2004). Clarifying the construct of mindfulness in the context of emotion regulation and the process of change in therapy. *Clinical Psychology: Science and Practice*, *11*, 255-262.

**G**,

Music selection for controlled pauses

The music was selected on the basis of the ratings from 12 young adults (none of whom participated later in the experiment). They rated their emotional states on a 1-5 scale, where 1 represented the negative emotion and 5 represented the positive emotion. Three samples out of 12 were selected for the study, the average rating of which being between 3 and 3.5. This procedure does not assure absolute neutrality, but excludes the possibility of inducing strong emotions and it provides more control over the participants’ resting behavior than a silent pause.

**H**,

**Additional results of Experiment III**

*Affectivity changes during the six TOJ threshold measurements*

- **Positive affectivity: t(39) = 4.303, p < .001 (decreased)**
- Upset: t(39) = -2.467, p = .018 (increased)
- Strong: t(39) = 2.399, p = .021 (decreased)
- **Enthusiastic: t(39) = 4.201, p < .001 (decreased)**
- Alert: t(39) = 2.687, p = .011 (decreased)
- Ashamed: t(39) = -2.223, p = .032 (increased)
- Inspired/Dedicated: t(39) = 2.481, p = .018 (decreased)
- Determined: t(39) = 3.365, p = .002 (decreased)
- Attentive: t(39) = 2.687, p = .011 (decreased)
- Active: t(39) = 2.926, p = .006 (decreased)
- Afraid: t(39) = 2.243, p = .031 (decreased)

Bonferroni threshold: .001923

*Affectivity changes during the six Easy TOJ threshold measurements*

- **Positive affectivity: t(39) = 2.585, p = .014 (decreased)**
- Interested: t(39) = 2.467, p = .018 (decreased)
- Scared: t(39) = 2.082, p = .044 (decreased)
- Inspired/Dedicated: t(39) = 2.511, p = .016 (decreased)
- Attentive: t(39) = 2.926, p = .006 (decreased)
- Afraid: t(39) = 2.211, p = .033 (decreased)

Bonferroni threshold: .001923

*Supplementary Table 11. Descriptive statistics of PANAS before and after the six TOJ threshold measurements*

| feeling | TOJ start group  pre | SD | ETOJ start  group pre | SD | TOJ start group post | SD | ETOJ start group post | SD |
| --- | --- | --- | --- | --- | --- | --- | --- | --- |
| Interested | **4.45** | .51 | **3.45** | .94 | **4.15** | .74 | **3.30** | .97 |
| Distressed | **1.05** | .22 | **1.00** | 0 | **1.30** | .65 | **1.10** | .30 |
| Excited | **3.25** | .91 | **2.5** | 1.19 | **3.55** | .88 | **2.30** | 1.26 |
| Upset | **1.05** | .22 | **1.05** | .22 | **1.45** | .60 | **1.10** | .30 |
| Strong | **3.15** | .933 | **3.1** | 1.02 | **2.95** | .88 | **2.70** | 1.26 |
| Guilty | **1.1** | .30 | **1.25** | .91 | **1.10** | .30 | **1.25** | .71 |
| Scared | **1.3** | .47 | **1.00** | 0 | **1.30** | .73 | **1.10** | .30 |
| Hostile | **1.05** | .22 | **1.00** | 0 | **1.00** | 0 | **1.05** | .22 |
| Enthusiastic | **4.05** | .82 | **3.45** | .99 | **3.65** | .87 | **2.95** | .94 |
| Proud | **2.60** | .88 | **2.55** | 1.31 | **2.35** | .98 | **2.45** | 1.23 |
| Bored | **1.05** | .22 | **1.45** | .68 | **1.30** | .65 | **1.50** | .36 |
| Irritable | **1.35** | .58 | **1.10** | .30 | **1.40** | .59 | **1.15** | .36 |
| Alert | **3.75** | .44 | **3.15** | .74 | **3.55** | .68 | **2.85** | .93 |
| Ashamed | **1.00** | 0 | **1.00** | 0 | **1.25** | .55 | **1.05** | .22 |
| Inspired | **3.4** | .94 | **3.35** | 1.08 | **3.05** | 1.14 | **3.05** | 1.19 |
| Nervous | **1.3** | .47 | **1.10** | .30 | **1.35** | .58 | **1.10** | .30 |
| Determined | **3.65** | .81 | **3.55** | 1.09 | **3.25** | 1.16 | **3.20** | 1.15 |
| Attentive | **4.15** | .48 | **3.6** | .82 | **3.75** | .55 | **3.50** | 1.05 |
| Jittery/Tense | **1.40** | .59 | **1.15** | .36 | **1.60** | .59 | **1.30** | .47 |
| Active | **3.95** | .68 | **3.15** | .67 | **3.60** | .82 | **2.90** | .91 |
| Afraid | **1.85** | .98 | **1.10** | .30 | **1.45** | .75 | **1.10** | .30 |
| Fatigued | **2.25** | .786 | **3.00** | 1.02 | **2.20** | 1.05 | **3.10** | 1.16 |

*Supplementary Table 12. Correlations with Specific Subjective Fatigue*

|  | variable | rho | p |
| --- | --- | --- | --- |
| 1 | Mean TOJ threshold | .238 | .140 |
| 2 | **Minimum TOJ threshold** | **.474** | **.002** |
| 3 | Maximum TOJ threshold | .184 | .257 |
| 4 | TOJ-trend | -.114 | .483 |
| 5 | Mean accuracy in Easy TOJ | -.134 | .410 |
| 6 | Worst accuracy in Easy TOJ | -.164 | .312 |
| 7 | **Best accuracy in Easy TOJ** | **-.332** | **.036** |
| 8 | EasyTOJ-trend | .075 | .647 |
| 9 | Max-Min TOJ | -.172 | .288 |
| 10 | **Perceived effort during the TOJ tasks** | **.393** | **.012** |
| 11 | **Perceived difficulty of the TOJ task** | **.418** | **.007** |
| 12 | **Change in positive affectivity (post-pre)** | **-.346** | **.029** |

Interpretation:

- Better minimal TOJ threshold is linked with less subjective fatigue.
- More accurate performance in the easy TOJ is kinked with more subjective fatigue.
- More perceived effort is linked with more fatigue.
- The task perceived as more difficult is linked with more fatigue.
- More decrease in positive affectivity is linked with more fatigue.

*Supplementary Table 13. Perceived effort and difficulty*

|  | **Effort during TOJ** | **Difficulty of TOJ** | **Effort during Easy TOJ** | **Difficulty of Easy TOJ** |
| --- | --- | --- | --- | --- |
| Mean (SD) | 6.55 (1.73) | 6.67 (1.73) | 4.35 (1.74) | 3.57 (1.89) |

Correlation between effort and difficulty during TOJ: rho(38) = .839, p < .001

Correlation between effort and difficulty during Easy TOJ: rho(38) = .671, p < .001

*Accuracy change in the Easy TOJ task*


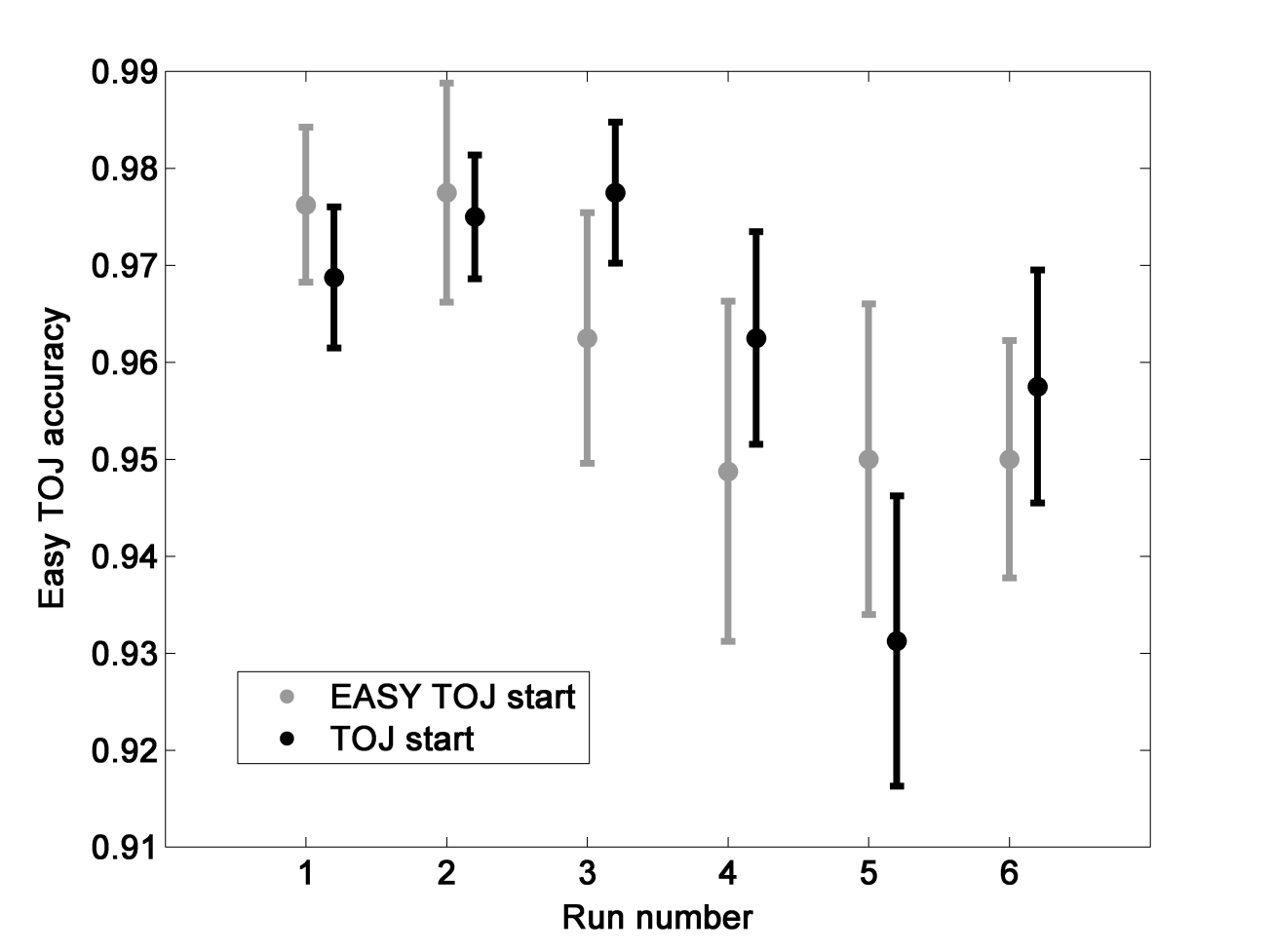


**Supplementary Fig. 9.** Performance deterioration during the EasyTOJ order judgement measurements, separately for the two groups of subjects. The error bar reflects the standard error.

*Supplementary Table 14. Descriptive statistics of the Achievement Goals Questionnaire*

|  | Mean | Std. Dev. |
| --- | --- | --- |
| Task-approach | **5.74** | 1.33 |
| Task-avoidance | **5.15** | 1.51 |
| Other-approach | **3.56** | 1.76 |
| Other-avoidance | **4.25** | 1.82 |

Table

*Supplementary Table 15. Task goals by group*

|  | Mean TOJ start | Std. Dev. | Mean EasyTOJ start | Std. Dev. |
| --- | --- | --- | --- | --- |
| Task-approach | **5.95** | .97 | **5.53** | 1.62 |
| Task-avoidance | **5.76** | 1.11 | **4.53** | 1.63 |
| Other-approach | **4.31** | 1.55 | **2.81** | 1.67 |
| Other-avoidance | **4.95** | 1.33 | **3.56** | 2.01 |
